# Supplementary material for: Tyrosine kinase targeting uncovers oncogenic pathway plasticity in Tasmanian devil transmissible cancers
Source: EMBO J. 2025 Nov 3;45(5):1426–59. doi: 10.1038/s44318-025-00603-0 (PMC12953634; doi:10.1038/s44318-025-00603-0)
Supplement: Supplementary file 1 — Appendix [file 44318_2025_603_MOESM1_ESM.pdf]

Appendix

EMBOJ-2025-120337 Schönbichler et al.

Tyrosine kinase targeting uncovers oncogenic pathway plasticity in Tasmanian devil transmissible cancers

Appendix Table of Content

Appendix Figure S1 .... Page 3

Appendix Figure S2 .... Page 5

Appendix Figure S3 .... Page 7

Appendix Figure S4 .... Page 9

Appendix Table S1 .... Page 11

Appendix Table S2 .... Page 12

Appendix Table S3 .... Page 13

Appendix Table S4 .... Page 14

Appendix Table S5.. Page 15

Appendix Figure S1 (Related to Figure 1)

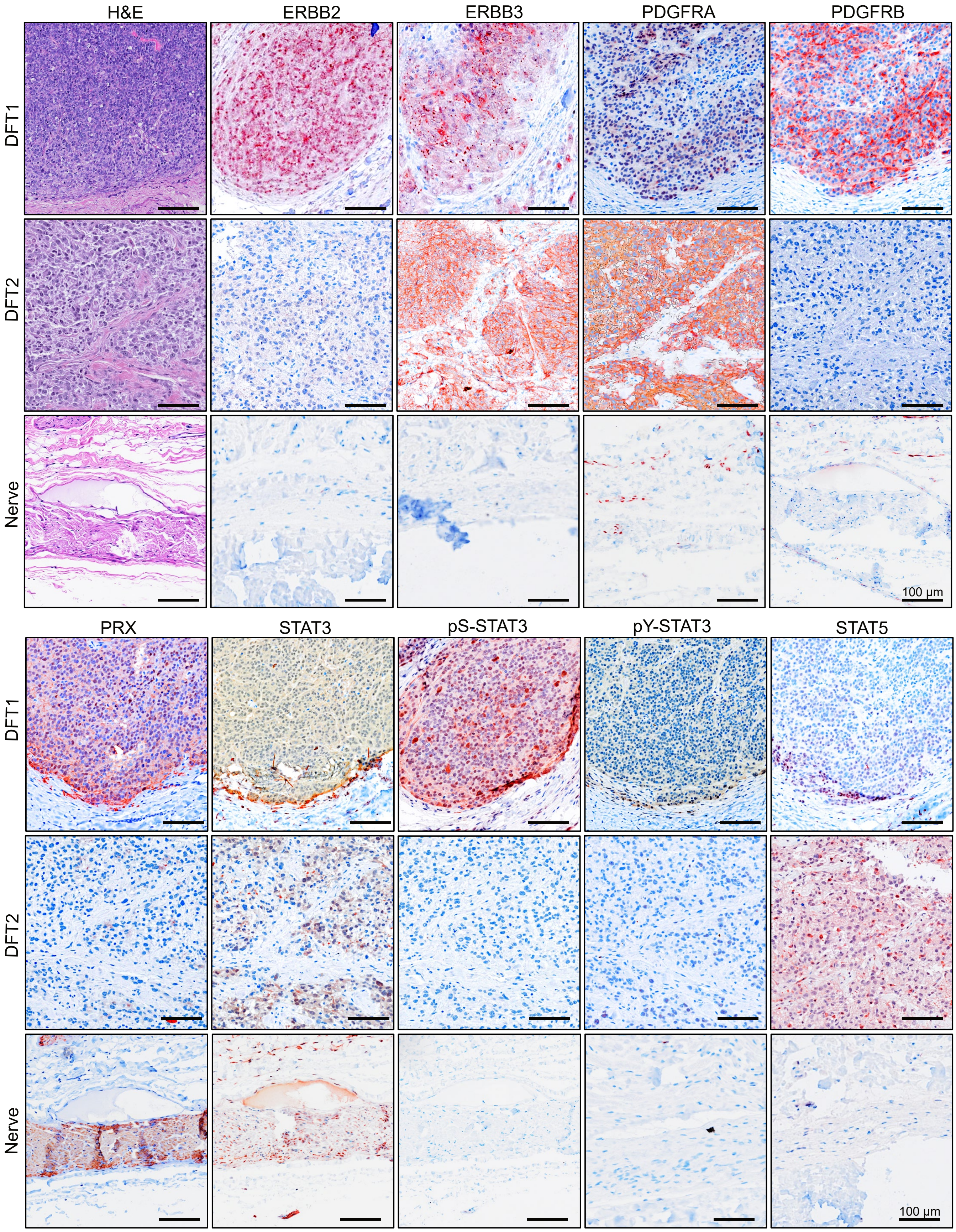

Appendix Figure S1 (Related to Figure 1)

Representative hematoxylin and eosin (H&E) staining and immunohistochemistry (IHC) for ERBB2, ERBB3, PDGFRα, PDGFRβ, PRX, total STAT3, phosphorylated STAT3 (pS-STAT3, pY-STAT3), and total STAT5. Consecutive sections are shown from a primary DFT1 tumour (T588), a primary DFT2 tumour (T607), and a peripheral nerve biopsy (Nerve 1). Scale bars, 100 µm. See **Appendix Table S2** for biopsy details.

Appendix Figure S2 (Related to Figure 2)

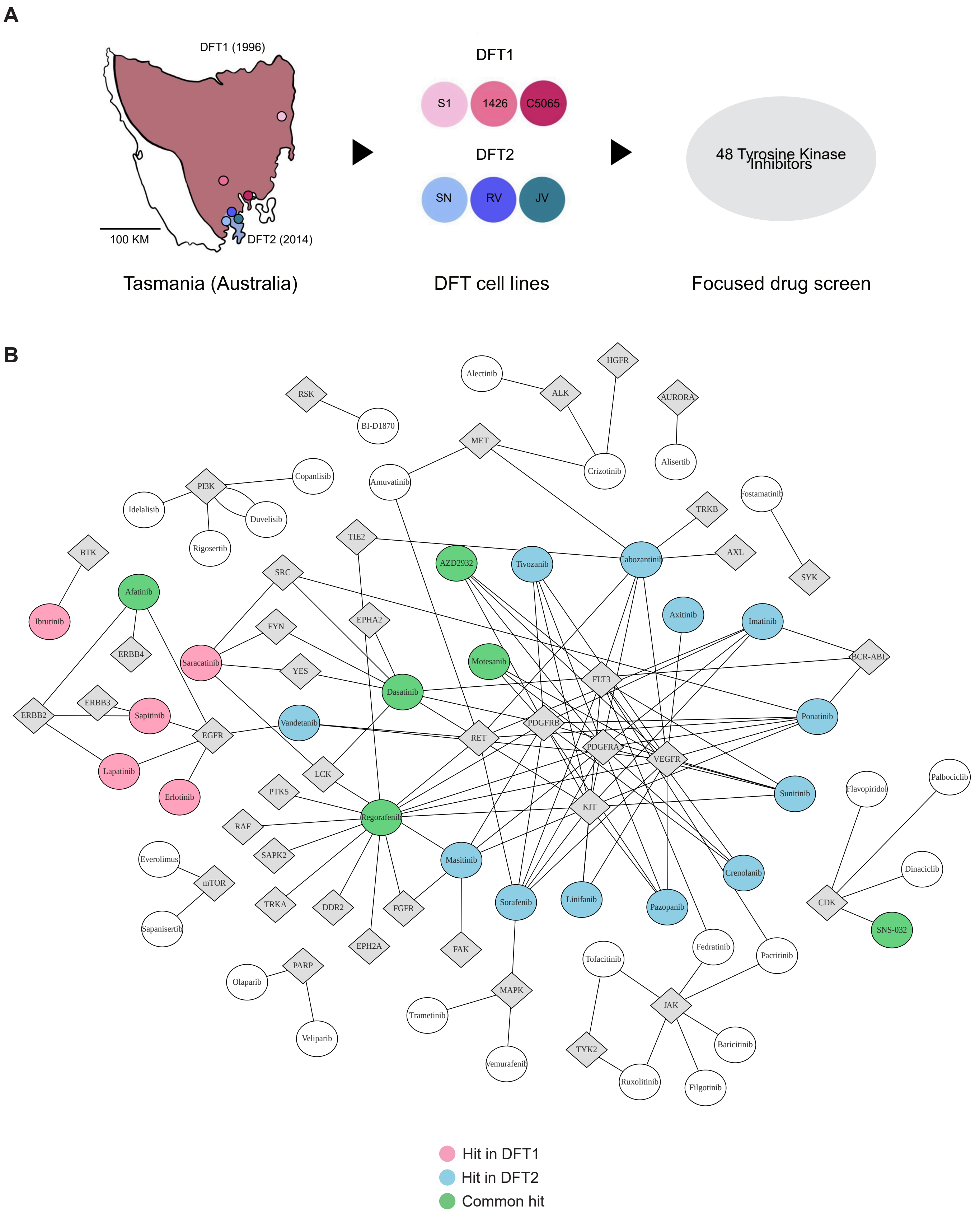

Appendix Figure S2 (Associated with Figure 2)

(A) Schematic of focused tyrosine kinase inhibitor (TKI) drug screen performed on three DFT1 (pink) and three DFT2 (blue) cell lines derived from tumour biopsies collected across various Tasmanian locations (map shown; see **Appendix Table S1**). A total of 48 TKIs were tested (see Source Data). A healthy fibroblast cell line served as a control.

(B) Network analysis of the 48 TKIs (ellipses), showing their primary targets (grey squares) as provided by compound vendors (see **Appendix Table S5**). Inhibitors are colour-coded by response: DFT1-specific hits (pink), DFT2-specific hits (blue), and common hits (green), as summarized in the Venn diagram (Figure 2A). Network visualisation was generated using Cytoscape Web (v1.0.3) (Shannon et al., 2003).

Appendix Figure S3 (Related to Figure 4)

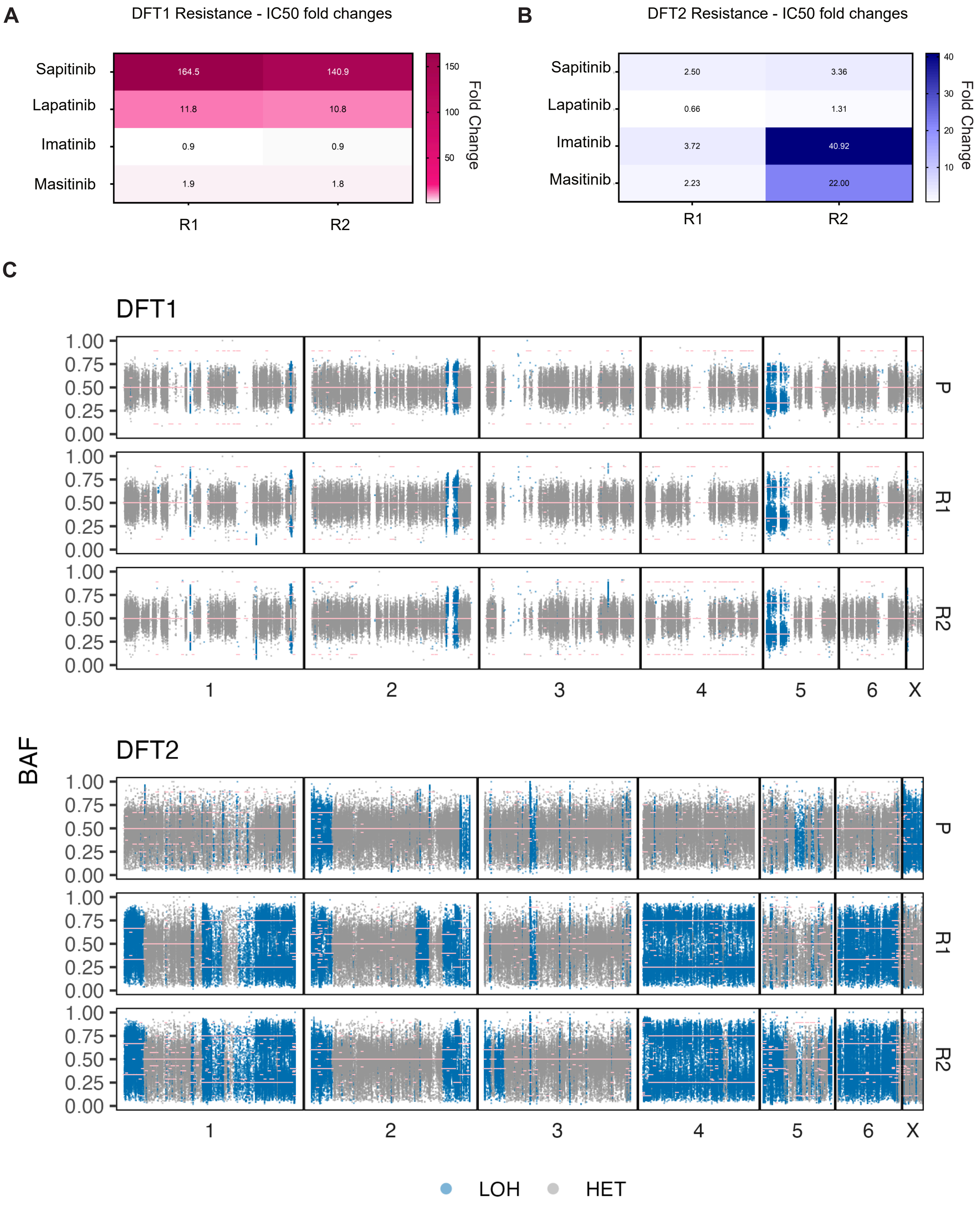

Appendix Figure S3 (Associated with Figure 4)

(A-B) Heatmaps of mean fold changes (n=3) of IC_{50} values for four selected drugs targeting ERBB or PDGFR in parental and two independent sapitinib-resistant DFT1 and two independent imatinib-resistant cell lines (R1/R2). (A) IC_{50} values were normalized to parental cell lines: sapitinib: 0.3 µM, lapatinib: 0.6 µM, imatinib: 25.8 µM, masitinib: 3.4 µM. (B) IC_{50} values were normalized to parental cell lines: sapitinib: 6.7 µM, lapatinib: 3.7 µM, imatinib: 0.1 µM, masitinib: 0.1 µM.

(C) B allele frequencies (BAFs) plots across chromosomes 1–6 and X for DFT1 and DFT2 parental cell lines and their resistant derivatives (R1 and R2). Pink dots indicate the A and B allele frequencies inferred by ControlFREEC over 50 kbp windows. Regions in blue have inferred A and B allele frequencies different from 0.5.

Appendix Figure S4 (Related to Figure 6)

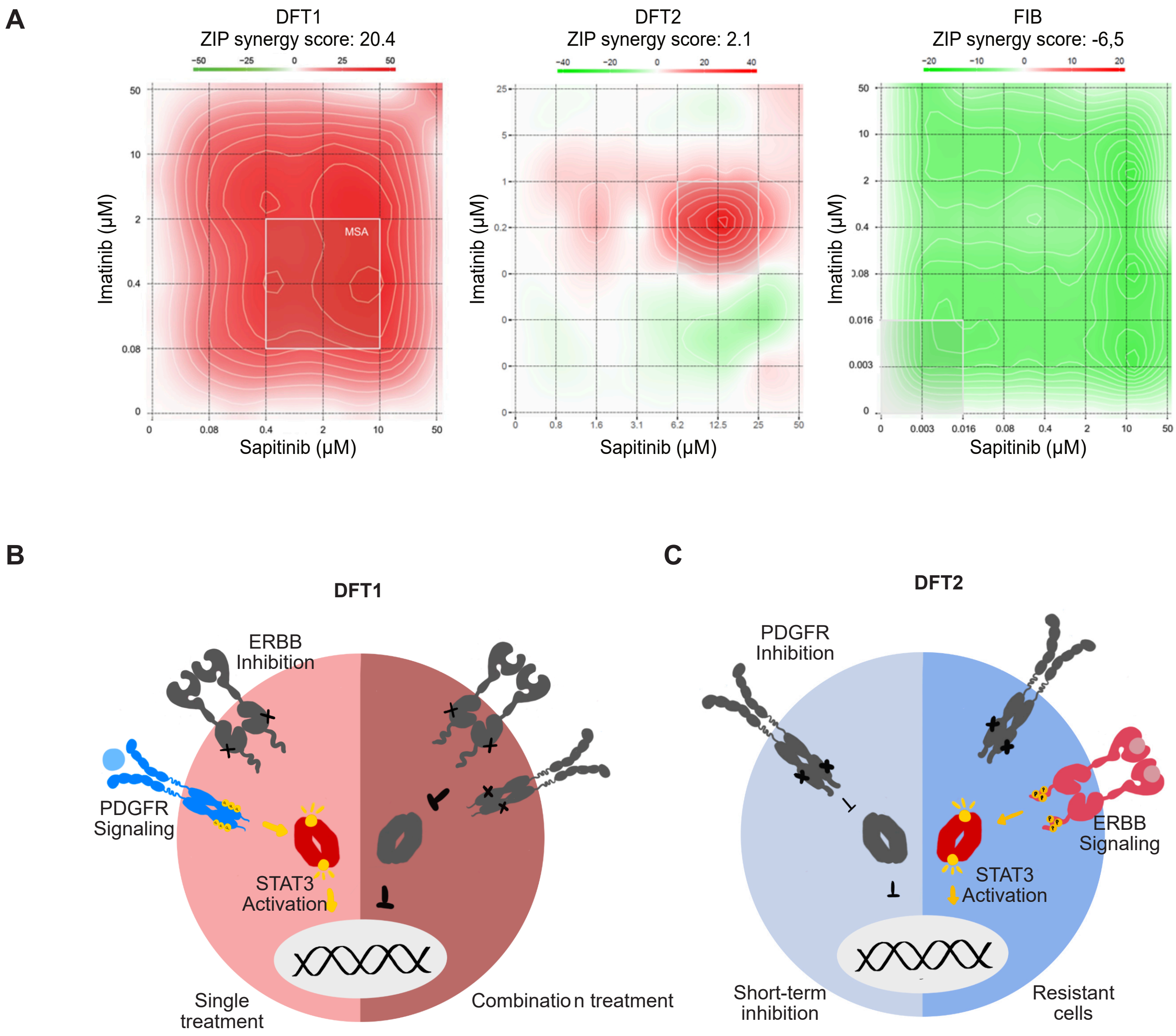

Appendix Figure S4 (Related to Figure 6)

(A) Representative synergy plots for sapitinib and imatinib combination treatment in DFT1, DFT2, and healthy fibroblast cell lines. Synergy assessed by SynergyFinder 3.0 with MSA >10 indicating synergy. Three independent experiments were performed.

(B) Illustrations of distinct signalling and resistance mechanisms in DFT1 and DFT2.

Appendix Table S1: Description of cell lines used in this study

Cell line	DFT	Accession #	Location	Sex	Age (Years)	Year of sampling	Reference
Strain 1	DFT1	06 / 2887	St Marys	F	1	2006	Deakin et al., PLoS Genet 2012
1426	DFT1	86T	Fentonbury	F	N/A	2005	Siddle et al., 2013
C5065	DFT1	87T	Forestier	N/A	N/A	2007	Siddle et al., 2013
Red Velvet (RV)	DFT2	202T2	Cygnet	M	3	2014	Pye et al., 2016b
Snug (SN)	DFT2	203T3	Snug	M	3	2014	Pye et al., 2016b
Jarvis (JV)	DFT2	338T	Coningham	M	2	2015	Pye et al., 2016b
Fibroblasts	Fibroblast cell line	91H	Taronga Zoo	F	N/A	2006	Murchison et al., Cell 2012

EMBOJ2025120337

Schönbichler et al.

Appendix Table S2: Description of biopsies used in this study

Devil	Sex	Age	Origin	Tumour	Year
T588	M	2 Years	Southport	DFT1	2017
T328		n.a.	n.a.	DFT1	n.a.
T603	M	2 Years	Elderslie	DFT1	2019
T607	M	2 Years	Pelverata	DFT2	2020
T594	M	5 Years	Woodbridge	DFT2	2018
T609	M	3 Years	Nicholls rivulet	DFT2	2021
Nerve 1	n.a.	n.a.	n.a.	Peripheral nerve	n.a.
TD269	F	n.a.	Triabunna	Peripheral nerve	2009

Appendix Table S3: Description of RT-qPCR primers used in this study

EGFR _forward	CTTCCTAAAAACCATCCAGGAGG	Kosack et al., 2019
EGFR _reverse	TTGGAGAGCACAGAAAGTGCAT	
ERBB2 _forward	TCTGGGGAACCCAAGTGTG	This study
ERBB2 _reverse	GTACAGGTGGCTCAGTGTGT	
ERBB3 _forward	TCCAGGGAGGATGTCCAGG	Kosack et al., 2019
ERBB3 _reverse	TTCCAGGTTTCCCATCACCA	
PDGFRA _forward	CGGAGTCACGTAGAGATAAGTTTTCC	Kosack et al., 2019
PDGFRA _reverse	TGGAAGCTGGCAAAATGTGA	
PDGFRB _forward	TGTGCCAGATTCCTCTGTGG	Kosack et al., 2019
PDGFRB _reverse	TCTCATGCAACGTTACCACCA	
STAT1 _forward	GGAAAAGCAAGACTGGGACTATGC	Kosack et al., 2019
STAT1 _reverse	GCGGCTATAGTGCTCATCCAA	
STAT3 _forward	AGGACTGGGCATATGCTGCC	Kosack et al., 2019
STAT3 _reverse	TGCTTGATTCTTCGCAGGTTGT	
STAT5B _forward	AGTTATGTGTGAATCTGCCAC	This study
STAT5B _reverse	GACAAACTCTGGGACCACCT	
MHCI _forward	CCGTGGGCTACGTGGACGATCAGC	Siddle et al., 2013
MHCI _reverse	GTCGTAGGCGAACTGAAG	
B2M _forward	TGTGCATCCTTCCCTACCTGGAGG	Siddle et al., 2013
B2M _reverse	CATTGTTGAAAGACAGATCGGACCGC	
TAP1 _forward	ACAGACTGGATCCTGCAGGATGAAG	Siddle et al., 2013
TAP1 _reverse	GAGACGTGATAGCACCTGTTTGG	
TAP2 _forward	TGTGGGCTAAGGCAGATTCTGG	Siddle et al., 2013
TAP2 _reverse	ATTCCCAGGAGGAGCTAAGCG	
RPL13A _forward	CCCCACAAGACCAAGCGAGGC	Siddle et al., 2013
RPL13A _reverse	ACAGCCTGGTATTTCCAGCCAACC	

EMBOJ2025120337
Schönbichler et al.
Appendix Table S4: Description of antibodies unsed in this study

**Antibody**	**Manufacturer**	**Catalog #**	**Clone**	**Species**	**Description of immunogen available information from commercial provider**
anti-PDGFR alpha	Cell Signaling Technology	5241	D13C6	rabbit monoclonal IgG	corresponding to the PDGF receptor α extracellular domain.
anti-PDGFR alpha	Abcam	ab124392	n.a.	rabbit polyclonal	immunogen corresponding to Synthetic Peptide within Human PDGFRA aa 1000 to C-terminus conjugated to Keyhole Limpet Haemocyanin.
anti-PDGFR alpha	Cell Signaling Technology	3174	D1E1E	rabbit monoclonal IgG	corresponding to residues near the carboxy-terminal sequence of human PDGFRα
anti-PDGFR beta	Cell Signaling Technology	8044	28E1	rabbit monoclonal IgG	monoclonal antibody is produced by immunizing animals with a GST fusion protein containing a carboxy-terminal fragment of human PDGF receptor β protein.
anti-PDGFR beta	Cell Signaling Technology	4564	C82A3	rabbit monoclonal IgG	monoclonal antibody is produced by immunizing animals with a GST fusion protein containing a carboxyl-terminal fragment of human PDGF receptor β.
anti-HER2/ErbB2	Cell Signaling Technology	4290	D8F12	rabbit monoclonal IgG	synthetic peptide corresponding to residues surrounding His422 of human HER2/ErbB2
anti-ERBB3	Cell Signaling Technology	12708	D22C5	rabbit monoclonal IgG	human ERBB3 residues surrounding Gln1109
anti-ERBB3	Cell Signaling Technology	4791	21D3	rabbit monoclonal IgG	detects endogenous HER3/ErbB3 proteins only when phosphorylated at tyrosine 1289.
anti-STAT3	BD Biosciences	610189	84/Stat3	mouse monoclonal IgG1	rat STAT3 1-175
anti-STAT3	Cell Signaling Technology	9139	124H6	mouse monoclonal IgG2a	synthetic peptide corresponding to residues surrounding Gln692 of human STAT3
anti-phospho-STAT3 (Tyr705)	Cell Signaling Technology	9131	n.a.	rabbit polyclonal	synthetic phosphopeptide corresponding to residues surrounding Tyr705 of mouse Stat3
anti-phospho-STAT3 (Ser727)	Cell Signaling Technology	9134	n.a	rabbit polyclonal	synthetic phosphopeptide corresponding to residues surrounding Ser727 of mouse Stat3
anti-STAT5	BD Biosciences	610191	Clone 89	mouse IgG2b	sheep Stat5 aa. 451-649
anti-periaxin	Sigma Aldrich	HPA001868	n.a.	rabbit polyclonal IgG	human
anti-SOX10	Abcam	ab180862	EPR4007-104	rabbit Recombinant Monoclonal	immunogen used to generate this antibody is proprietary information
anti-Ki-67	Cell Signaling Technology	12202	D385	rabbit monoclonal IgG	recombinant protein specific to the amino terminus of Ki-67 protein.
anti-vWF	Invitrogen Antibodies	PA5-16634	n.a.	rabbit polyclonal IgG	purified factor VIII related antigen/von Willebrand factor.
anti-cleaved caspase-3 (Asp175)	Cell Signaling Technology	9661	D175	rabbit polyclonal IgG	detects endogenous levels of the large fragment (17/19 kDa) of activated caspase-3 resulting from cleavage adjacent to Asp175.
anti-HSC70	Santa Cruz Biotechnology	sc-7298	B-6	mouse monoclonal IgG2a	human HSC 70 580-601

EMBOJ2025120337
Schönbichler et al.
Appendix Table S5: Sequencing statistics

ID	Cell line	Raw reads	Trimmed reads	type	duplication rate	avg. insert size (bp)	median coverage
347097 Run1	DFT1	944599386	941775360	150 bp PE	16%	160,2	15
347098 Run1	DFT1 R1	960982506	959227052	150 bp PE	16%	165,4	16
347099 Run1	DFT1 R2	999509756	996231692	150 bp PE	19%	197,1	18
347100 Run1	DFT2	971716966	967968482	150 bp PE	18%	186,1	17
347101 Run1	DFT2 R1	944919674	943768752	150 bp PE	18%	178,4	17
347102 Run1	DFT2 R2	985391454	983282536	150 bp PE	18%	173,3	17
347097 Run2	DFT1	986872572	984343812	150 bp PE	17%	495,9	27
347098 Run2	DFT1 R1	984563906	983493990	150 bp PE	15%	345,2	31
347099 Run2	DFT1 R2	1003357648	1001701728	150 bp PE	16%	365,9	31
347100 Run2	DFT2	977988512	976492098	150 bp PE	30%	570,9	28
347101 Run2	DFT2 R1	1157223860	1156769940	150 bp PE	31%	410,4	34
347102 Run2	DFT2 R2	1073605520	1072670390	150 bp PE	30%	457,6	31
347097 Merged	DFT1		1926119172	150 bp PE			42
347098 Merged	DFT1 R1		1942721042	150 bp PE			47
347099 Merged	DFT1 R2		1997933420	150 bp PE			49
347100 Merged	DFT2		1944460580	150 bp PE			46
347101 Merged	DFT2 R1		2100538692	150 bp PE			51
347102 Merged	DFT2 R2		2055952926	150 bp PE			48
