# Supplementary figures and images for: Tyrosine kinase targeting uncovers oncogenic pathway plasticity in Tasmanian devil transmissible cancers
Source: EMBO J. 2025 Nov 3;45(5):1426–59. doi: 10.1038/s44318-025-00603-0 (PMC12953634; doi:10.1038/s44318-025-00603-0)

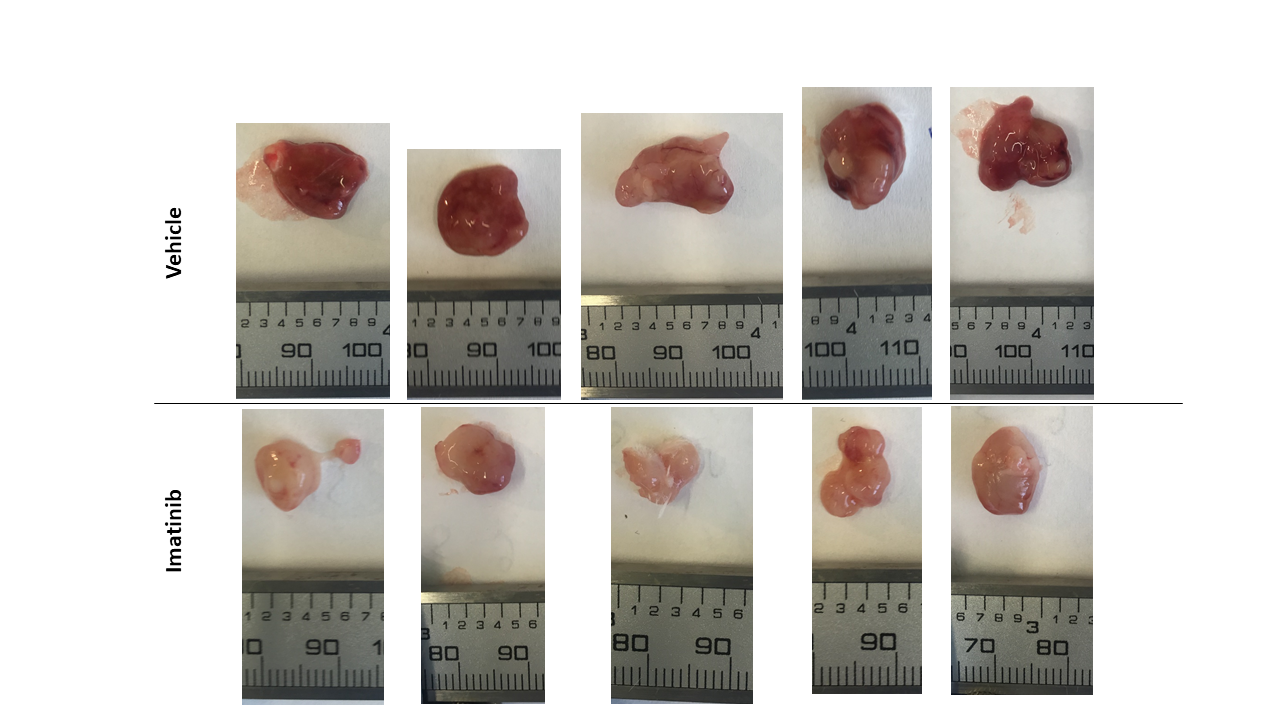

Supplement: Supplementary file 8 — Source data Fig. 3 [file 44318_2025_603_MOESM8_ESM.zip › EMBOJ2025120337_SourceData_Figure3/3D/DFT2_Xenograft_Imatinib_Tumour_Pictures.tif]

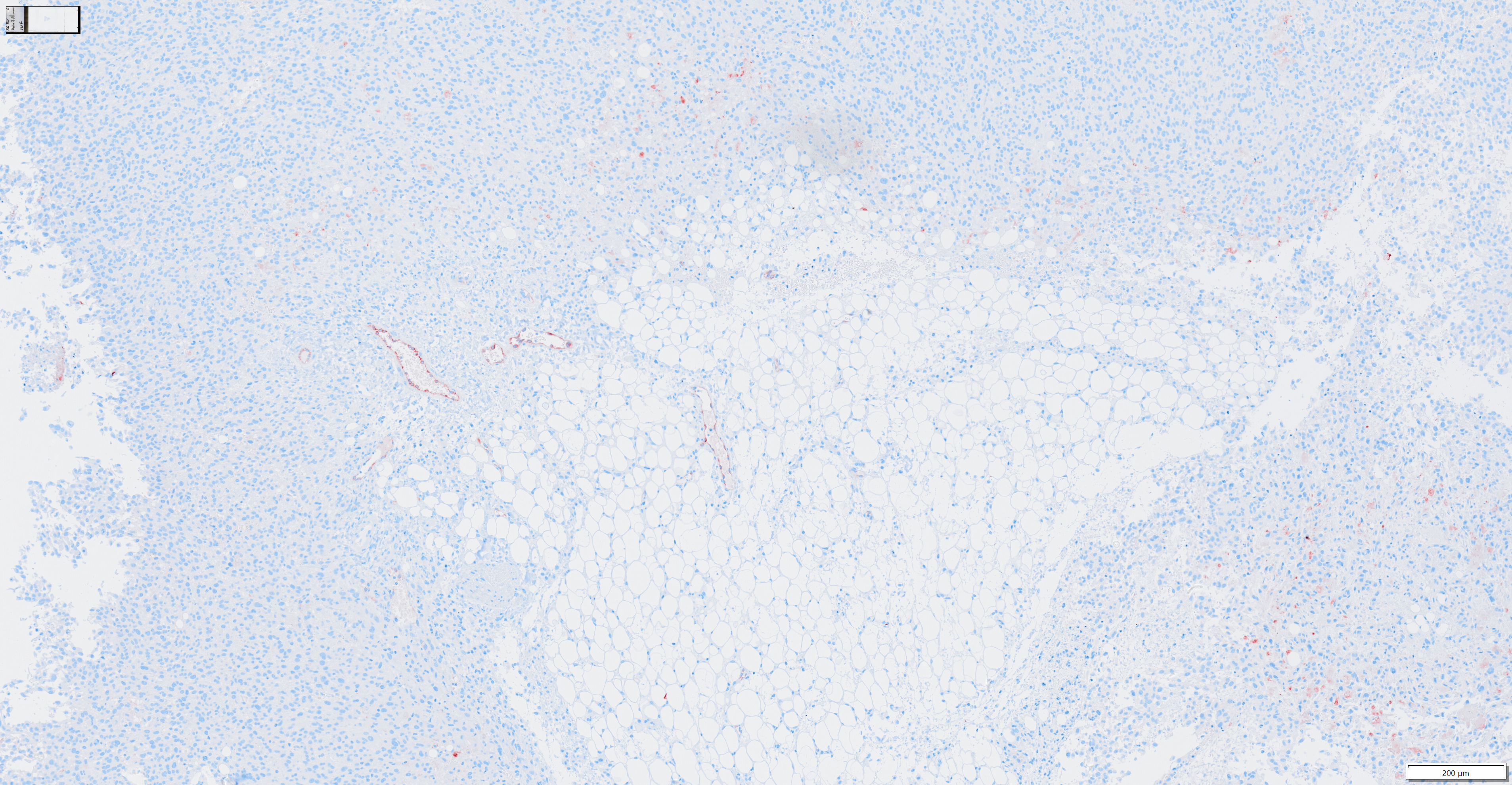

Supplement: Supplementary file 8 — Source data Fig. 3 [file 44318_2025_603_MOESM8_ESM.zip › EMBOJ2025120337_SourceData_Figure3/3H/Xenograft_Vehicle_VWF_200.tif]

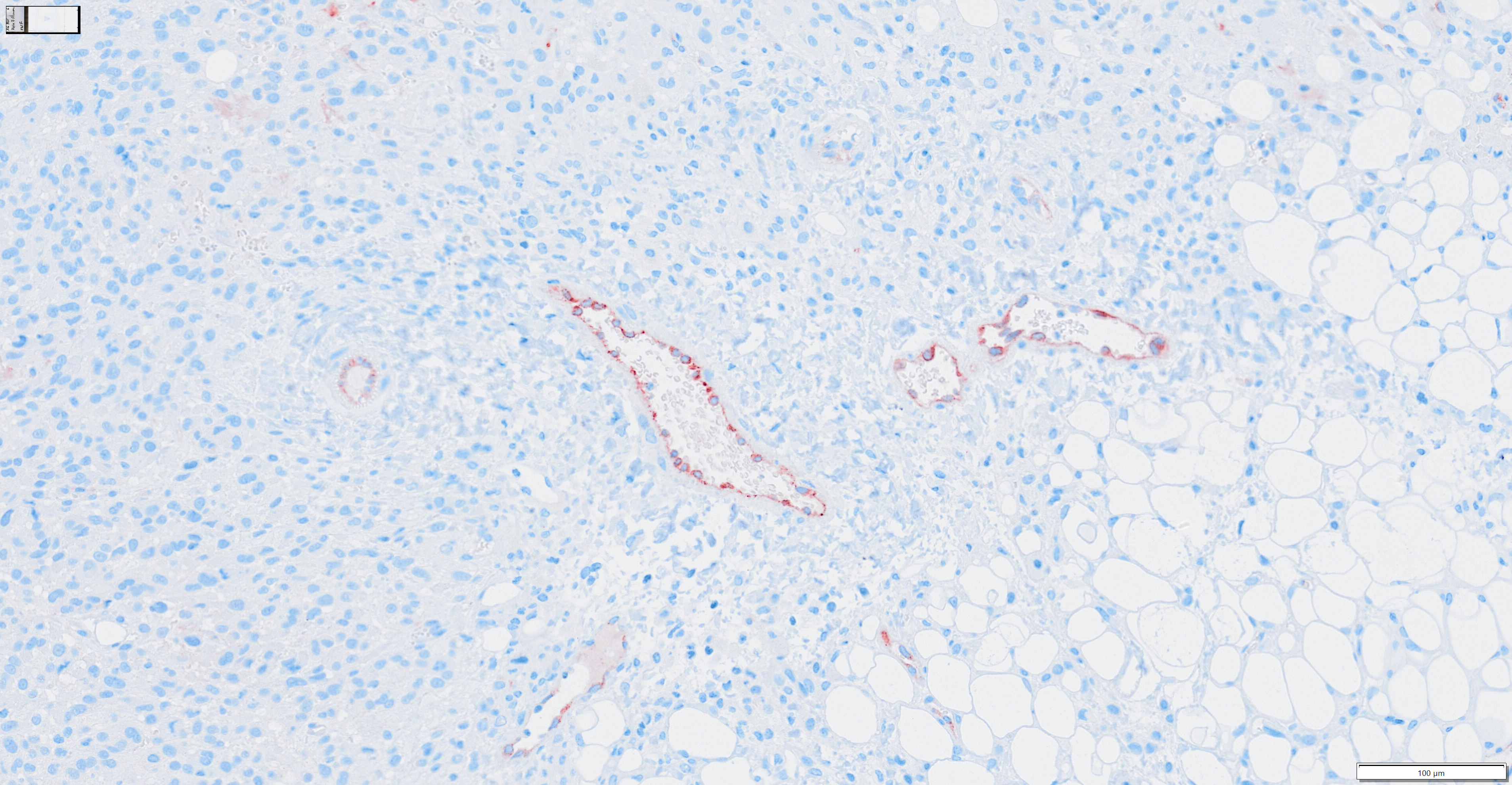

Supplement: Supplementary file 8 — Source data Fig. 3 [file 44318_2025_603_MOESM8_ESM.zip › EMBOJ2025120337_SourceData_Figure3/3H/Xenograft_Vehicle_VWF_100.tif]

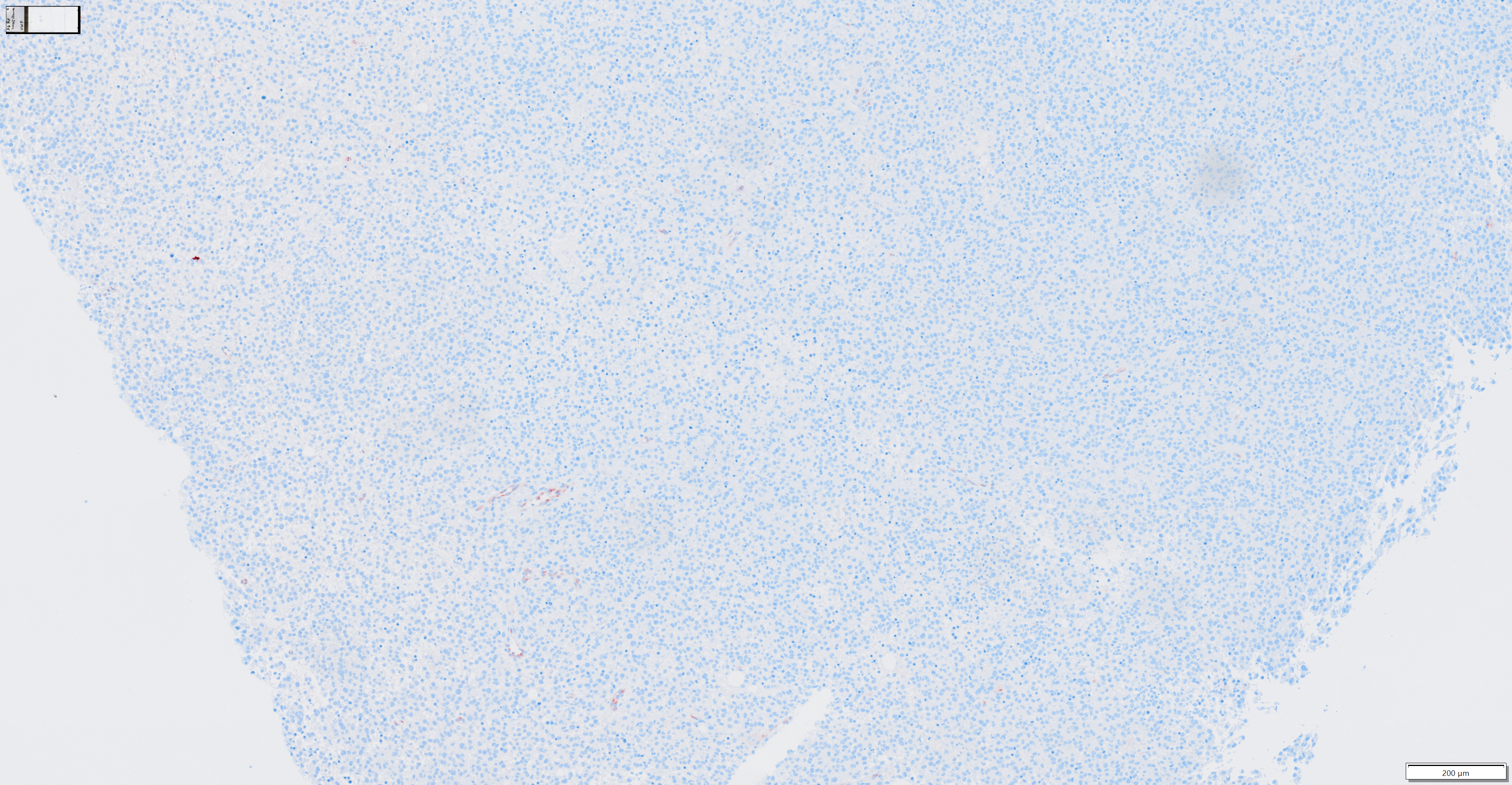

Supplement: Supplementary file 8 — Source data Fig. 3 [file 44318_2025_603_MOESM8_ESM.zip › EMBOJ2025120337_SourceData_Figure3/3H/Xenograft_Imatinib_VWF_200.tif]

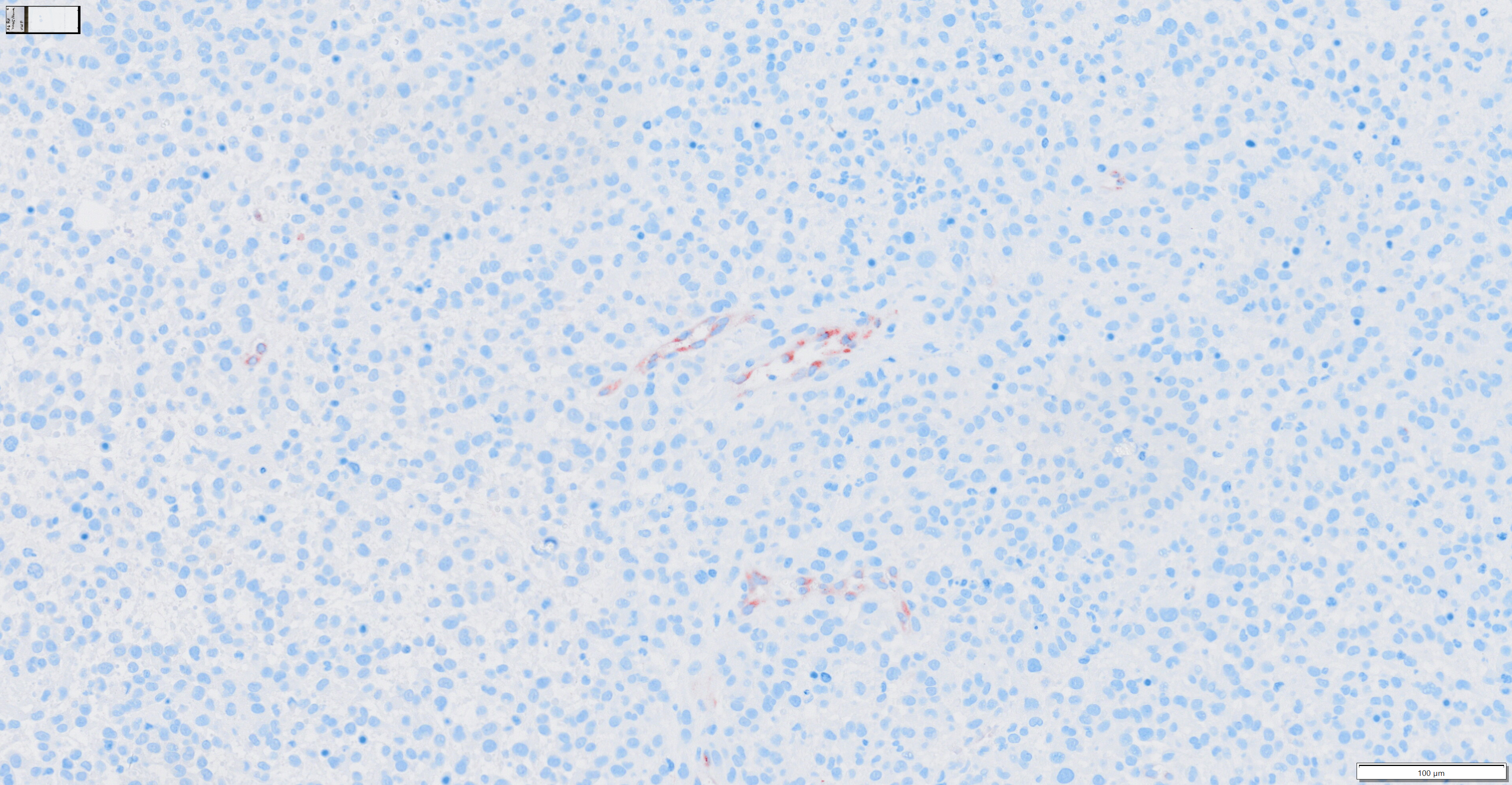

Supplement: Supplementary file 8 — Source data Fig. 3 [file 44318_2025_603_MOESM8_ESM.zip › EMBOJ2025120337_SourceData_Figure3/3H/Xenograft_Imatinib_VWF_100.tif]

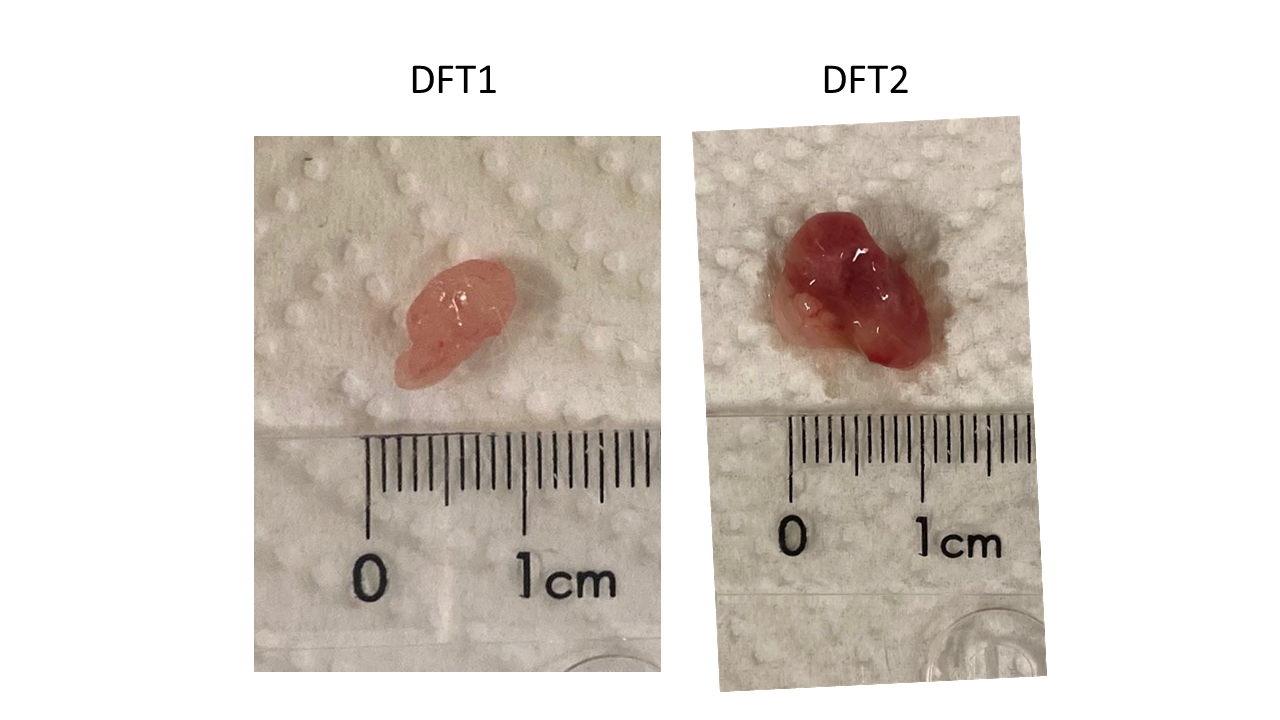

Supplement: Supplementary file 8 — Source data Fig. 3 [file 44318_2025_603_MOESM8_ESM.zip › EMBOJ2025120337_SourceData_Figure3/3A/Pictures_Tumours_DFT1_DFT2.tif]

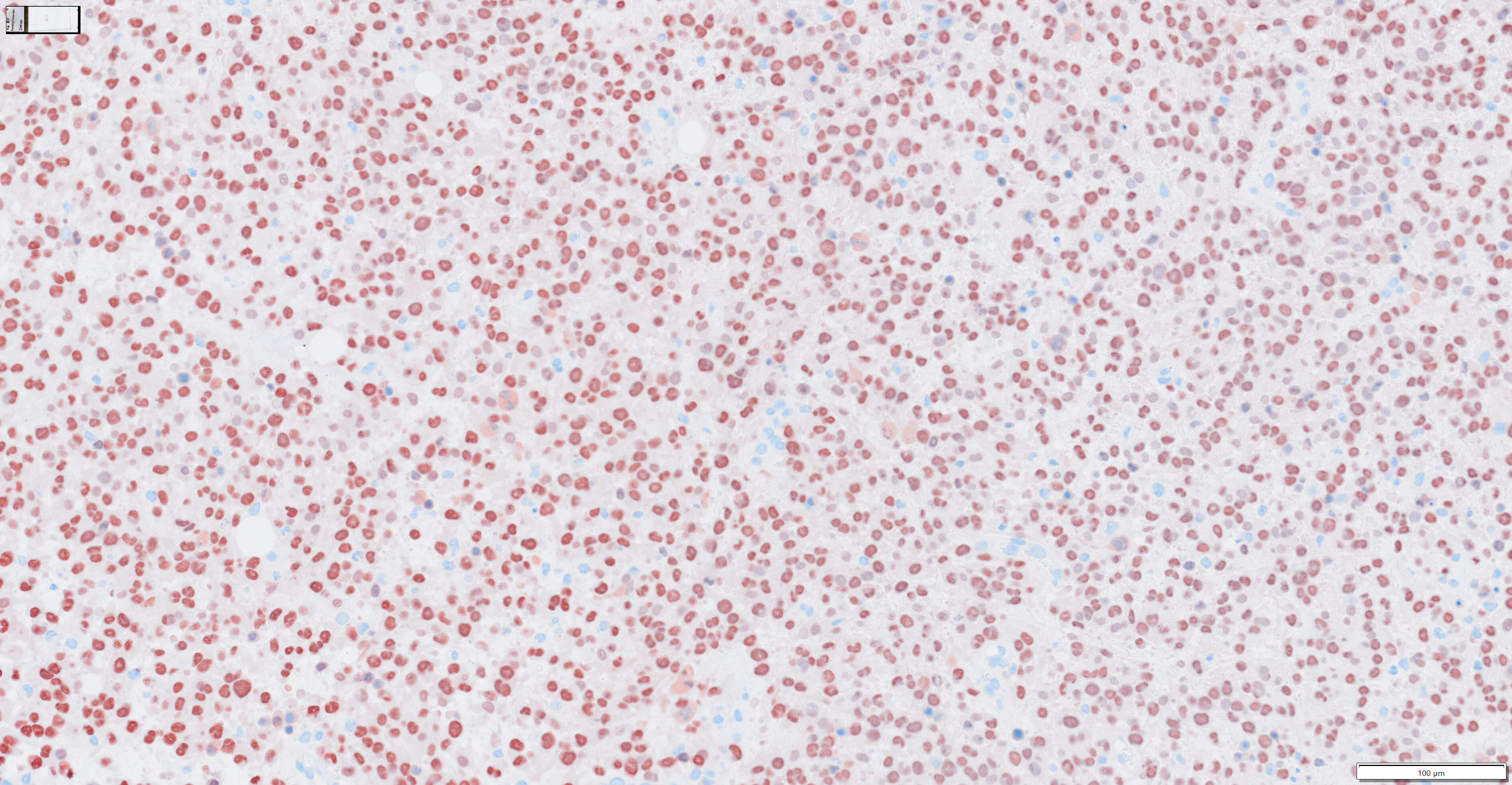

Supplement: Supplementary file 8 — Source data Fig. 3 [file 44318_2025_603_MOESM8_ESM.zip › EMBOJ2025120337_SourceData_Figure3/3F/Xenograft_Imatinib_SOX10_100.tif]

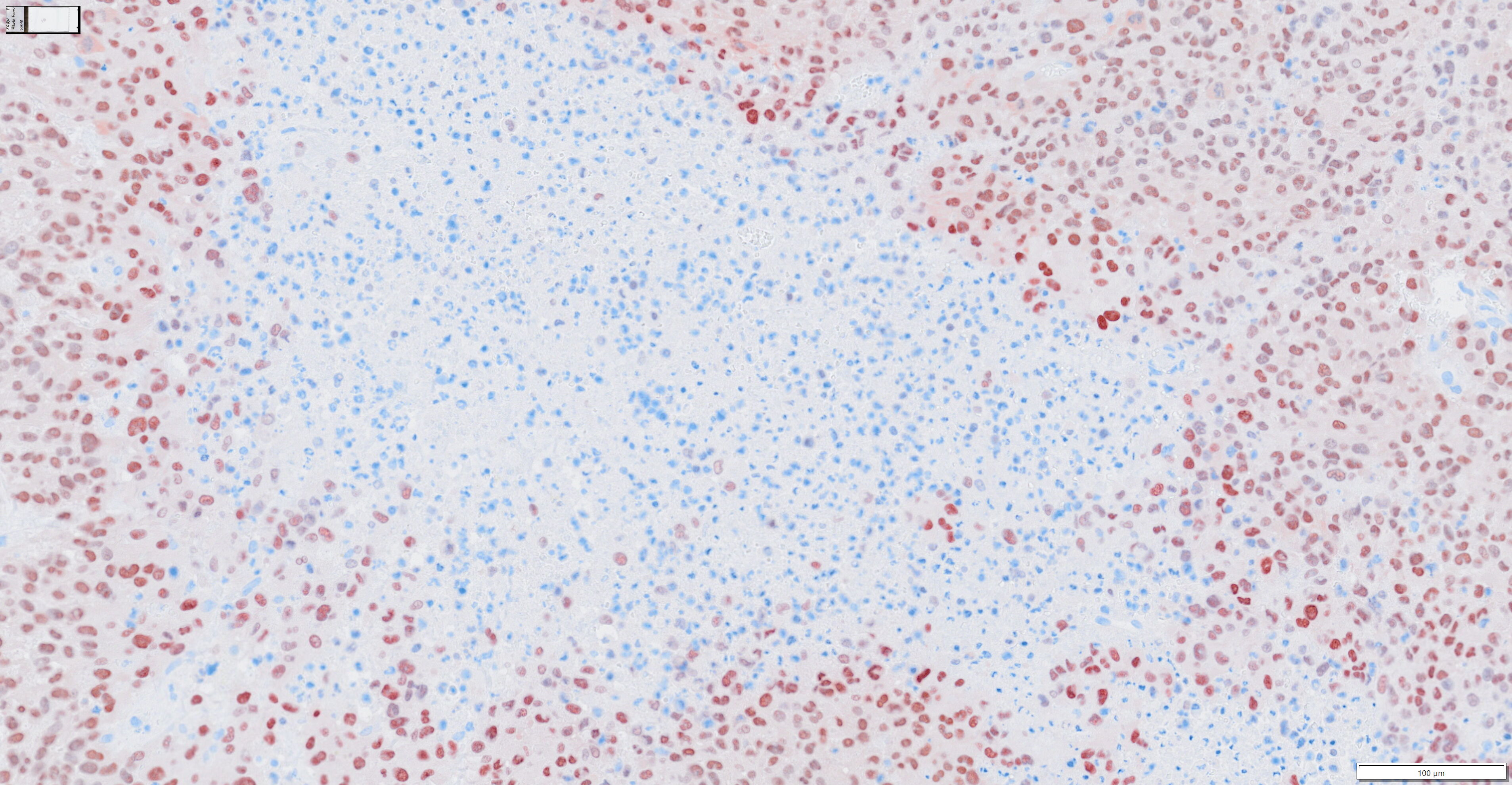

Supplement: Supplementary file 8 — Source data Fig. 3 [file 44318_2025_603_MOESM8_ESM.zip › EMBOJ2025120337_SourceData_Figure3/3F/Xenograft_Vehicle_SOX10_100.tif]

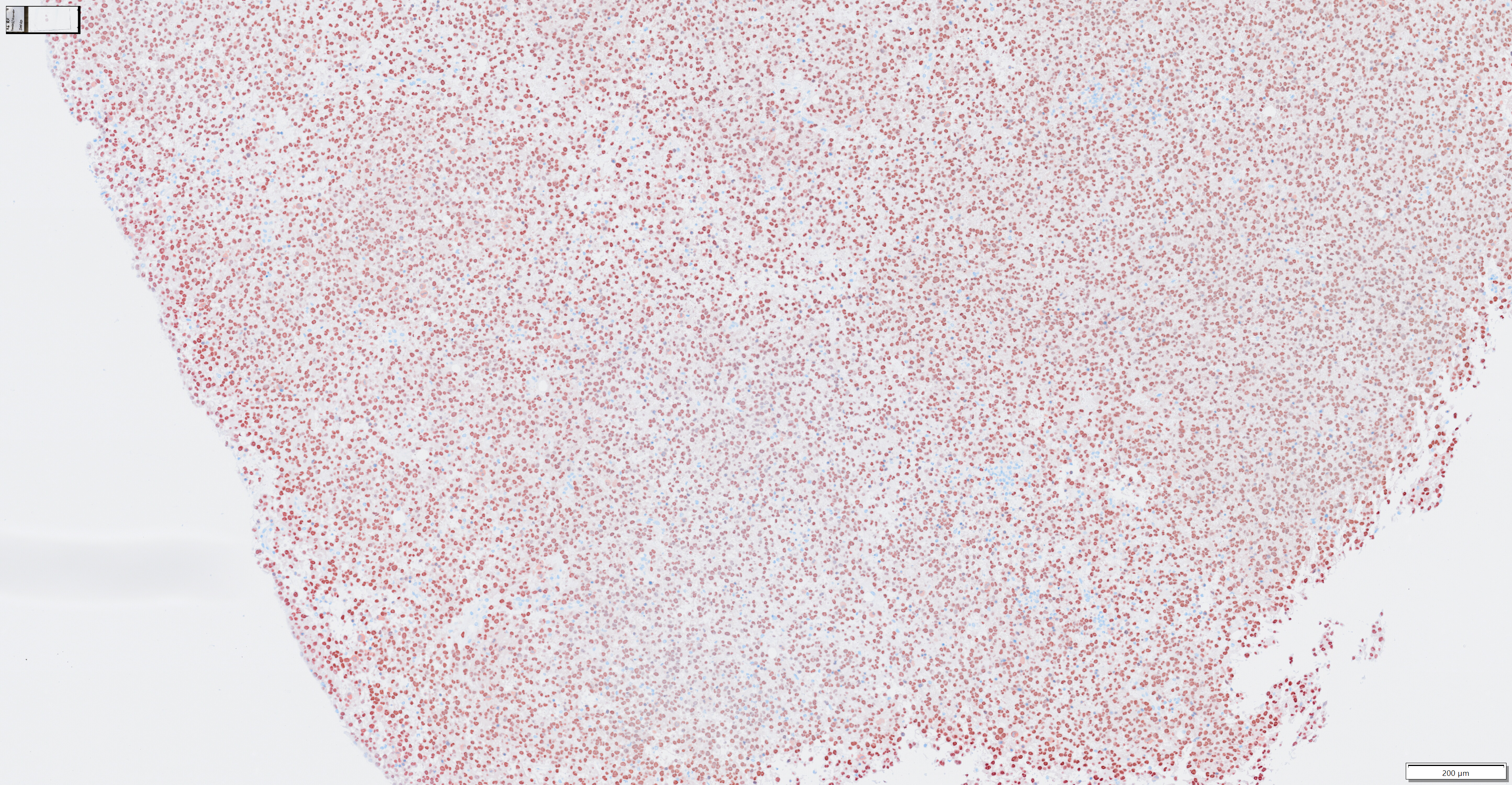

Supplement: Supplementary file 8 — Source data Fig. 3 [file 44318_2025_603_MOESM8_ESM.zip › EMBOJ2025120337_SourceData_Figure3/3F/Xenograft_Imatinib_SOX10_200.tif]

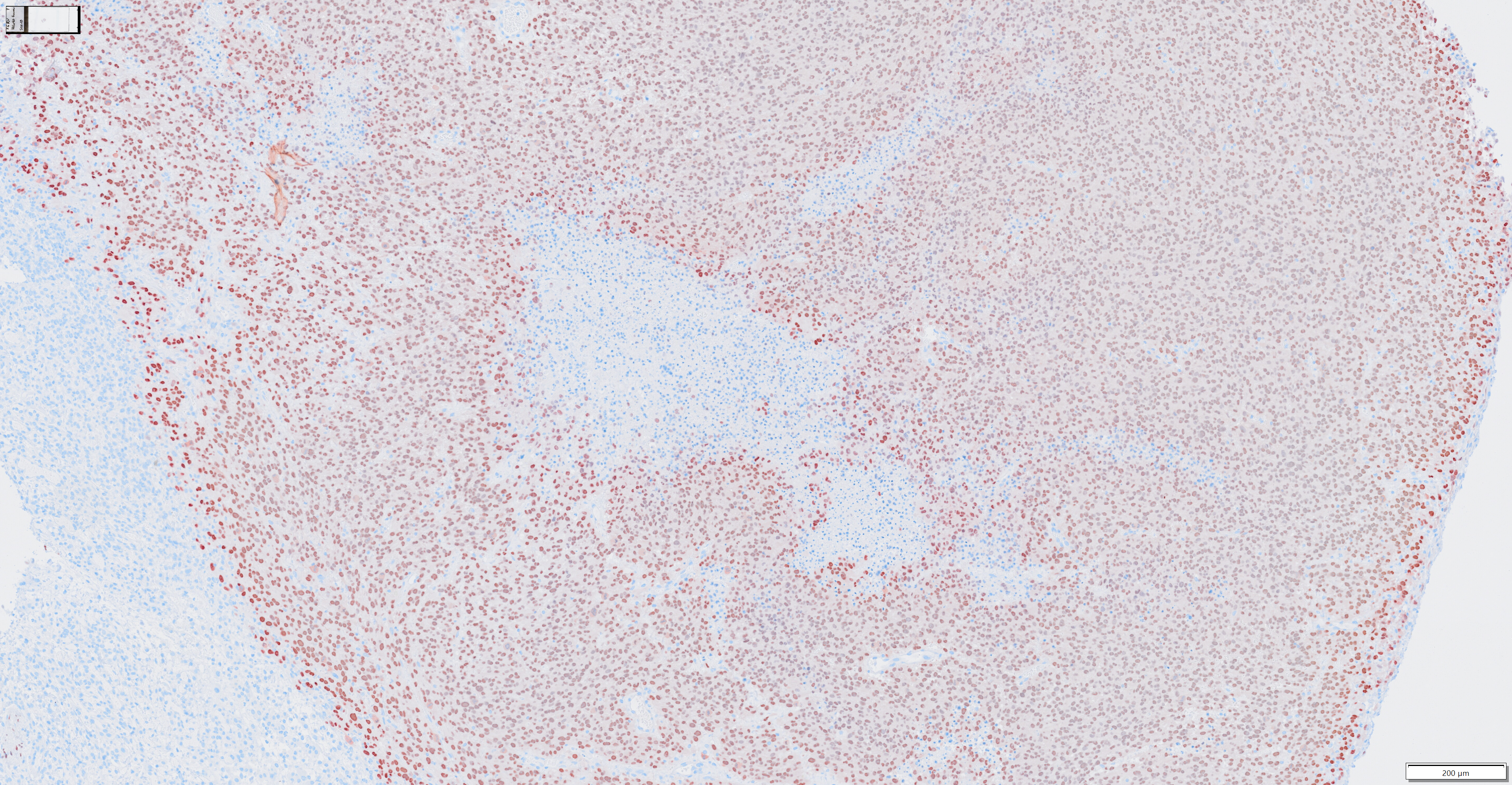

Supplement: Supplementary file 8 — Source data Fig. 3 [file 44318_2025_603_MOESM8_ESM.zip › EMBOJ2025120337_SourceData_Figure3/3F/Xenograft_Vehicle_SOX10_200.tif]

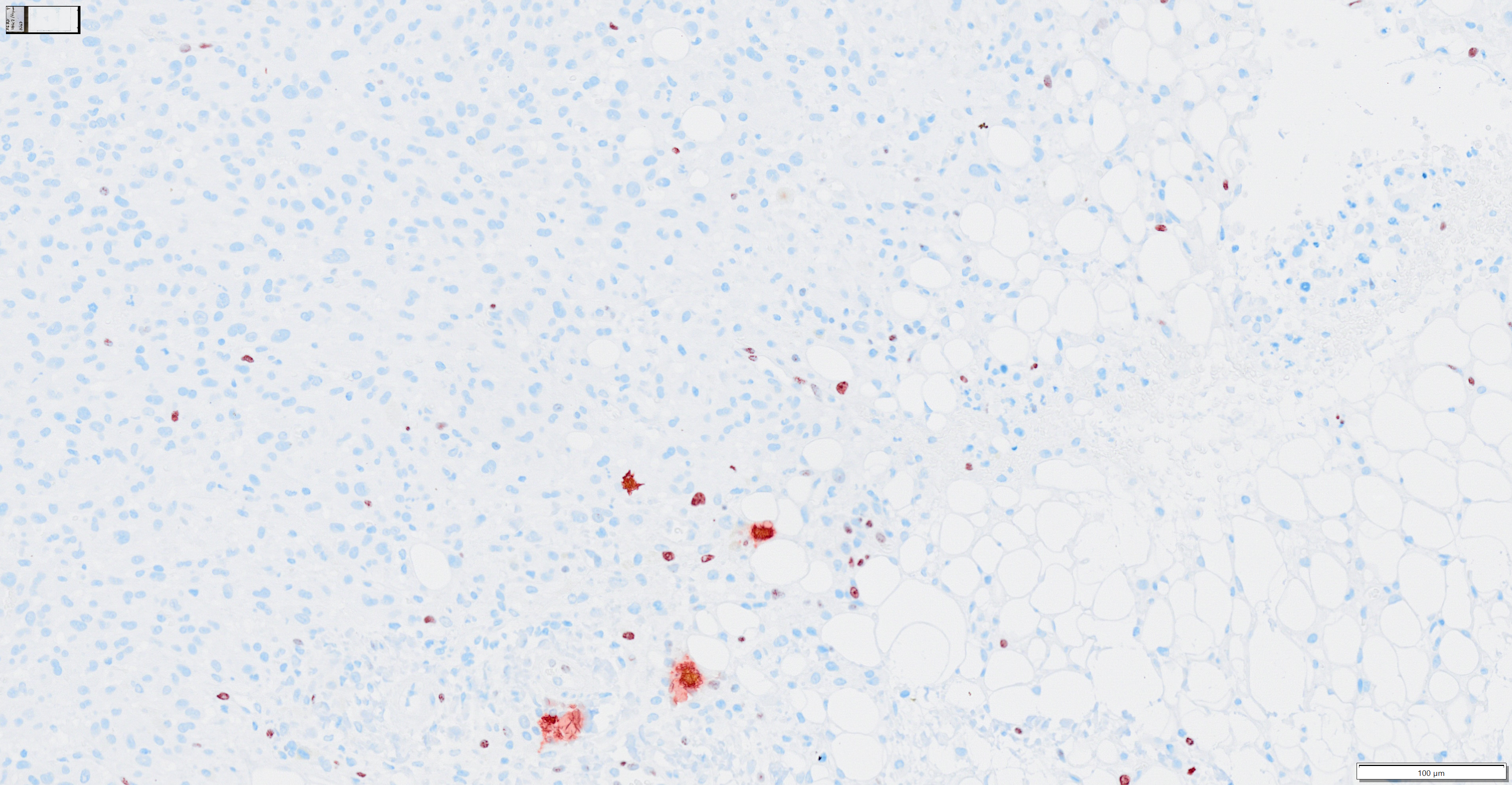

Supplement: Supplementary file 8 — Source data Fig. 3 [file 44318_2025_603_MOESM8_ESM.zip › EMBOJ2025120337_SourceData_Figure3/3G/Xenograft_Vehicle_Ki67_100.tif]

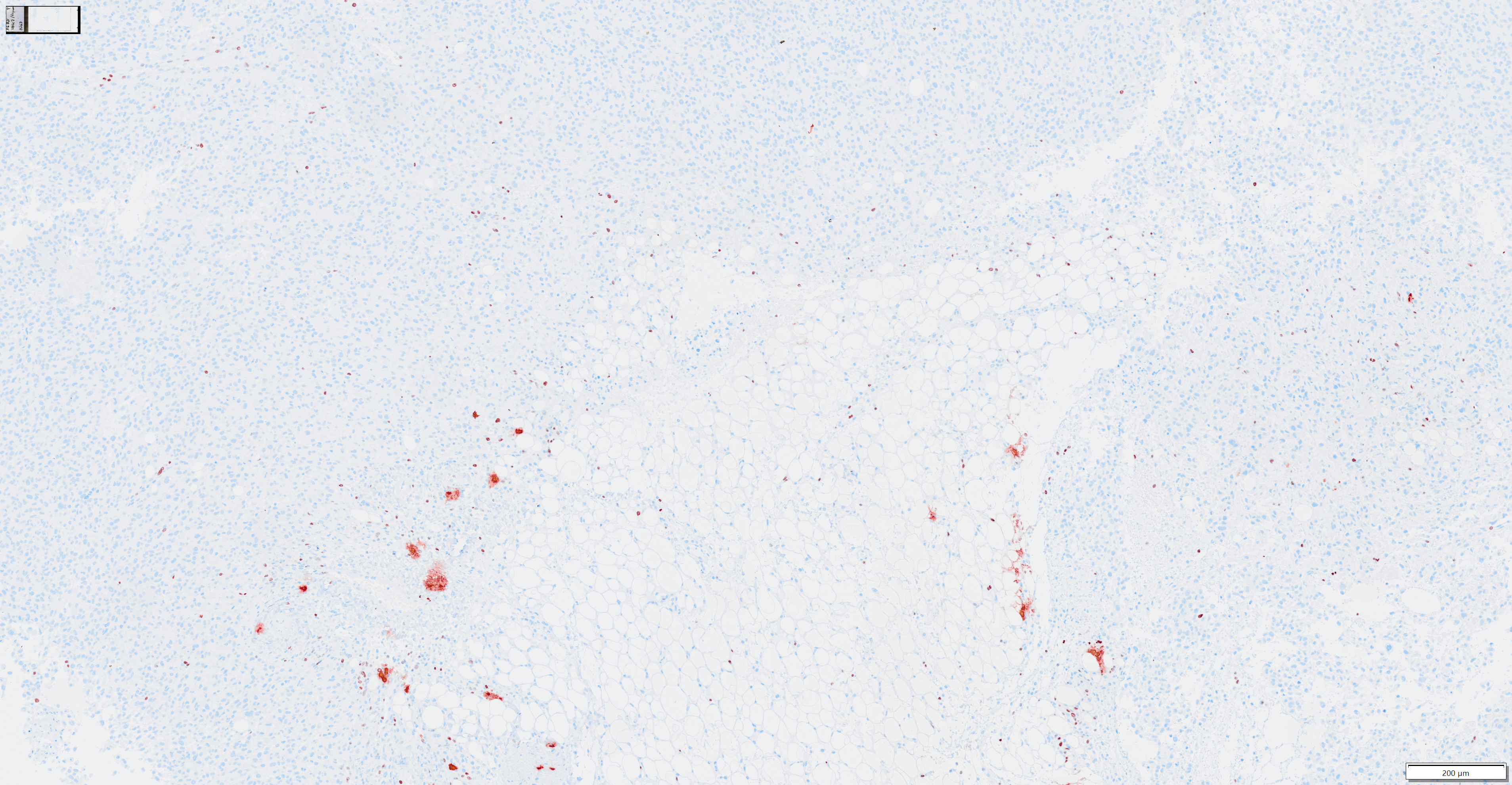

Supplement: Supplementary file 8 — Source data Fig. 3 [file 44318_2025_603_MOESM8_ESM.zip › EMBOJ2025120337_SourceData_Figure3/3G/Xenograft_Vehicle_Ki67_200.tif]

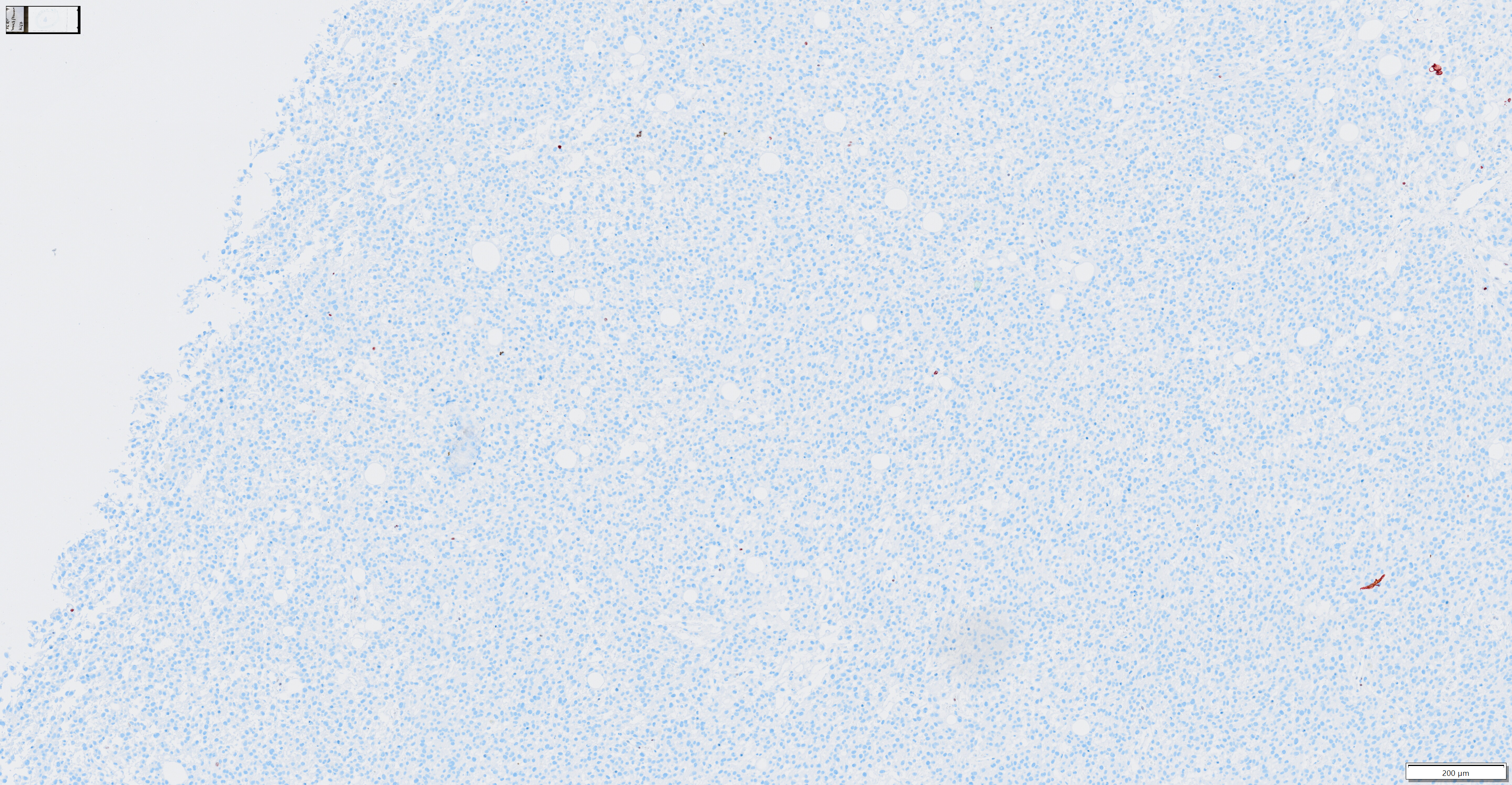

Supplement: Supplementary file 8 — Source data Fig. 3 [file 44318_2025_603_MOESM8_ESM.zip › EMBOJ2025120337_SourceData_Figure3/3G/Xenograft_Imatinib_Ki67_200.tif]

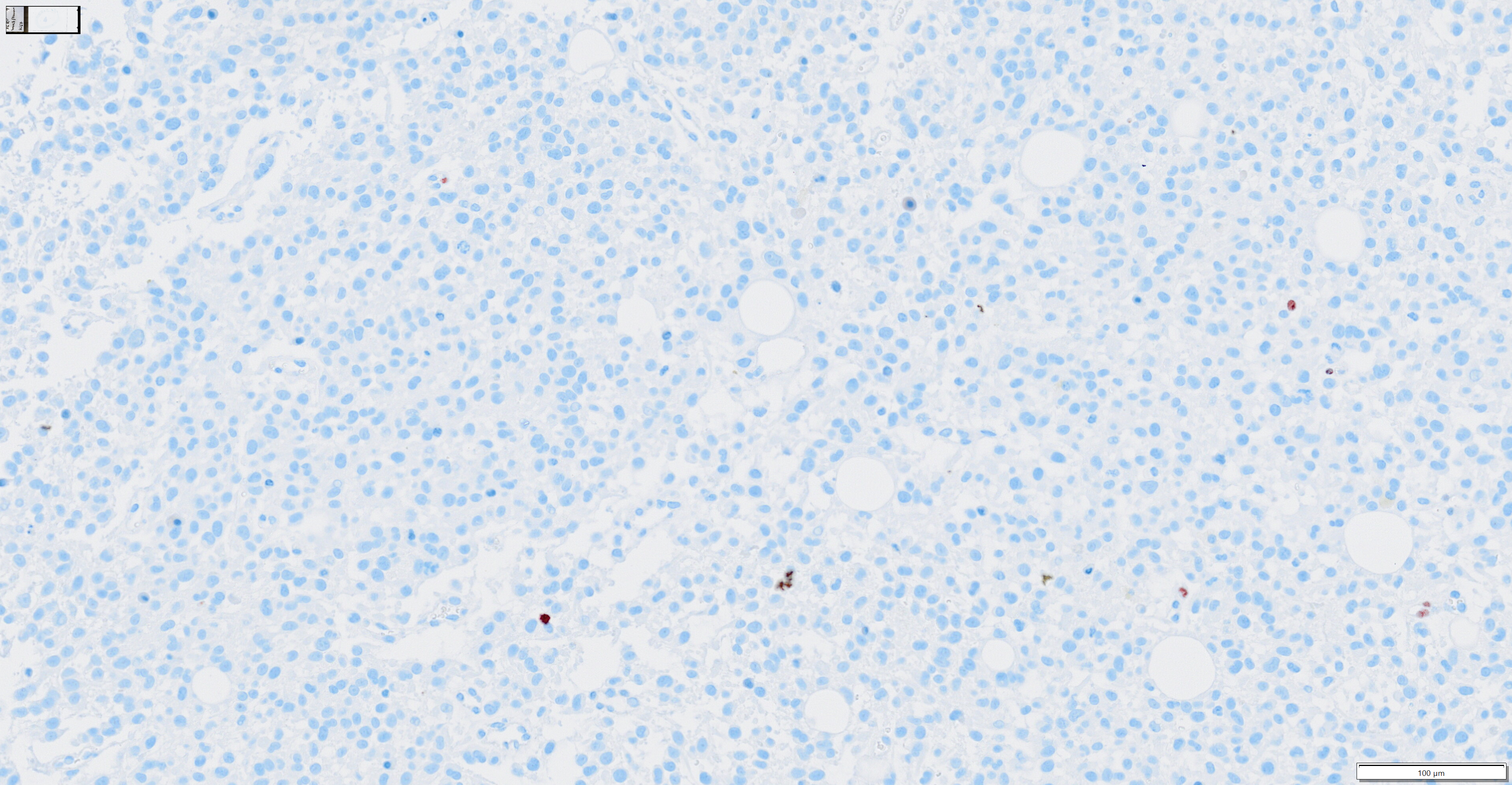

Supplement: Supplementary file 8 — Source data Fig. 3 [file 44318_2025_603_MOESM8_ESM.zip › EMBOJ2025120337_SourceData_Figure3/3G/Xenograft_Imatinib_Ki67_100.tif]

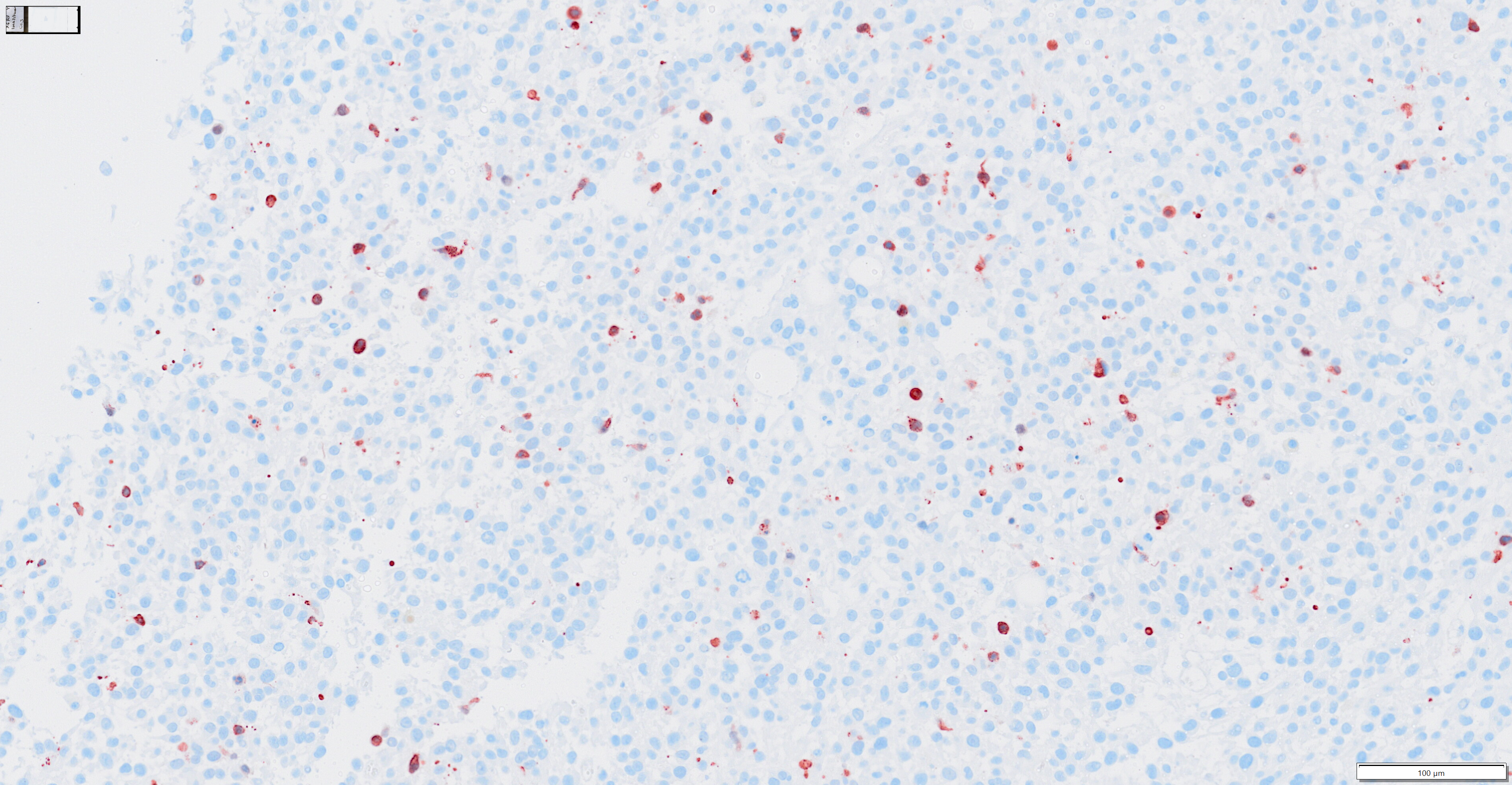

Supplement: Supplementary file 8 — Source data Fig. 3 [file 44318_2025_603_MOESM8_ESM.zip › EMBOJ2025120337_SourceData_Figure3/3I/Xenograft_Imatinib_CC3_100.tif]

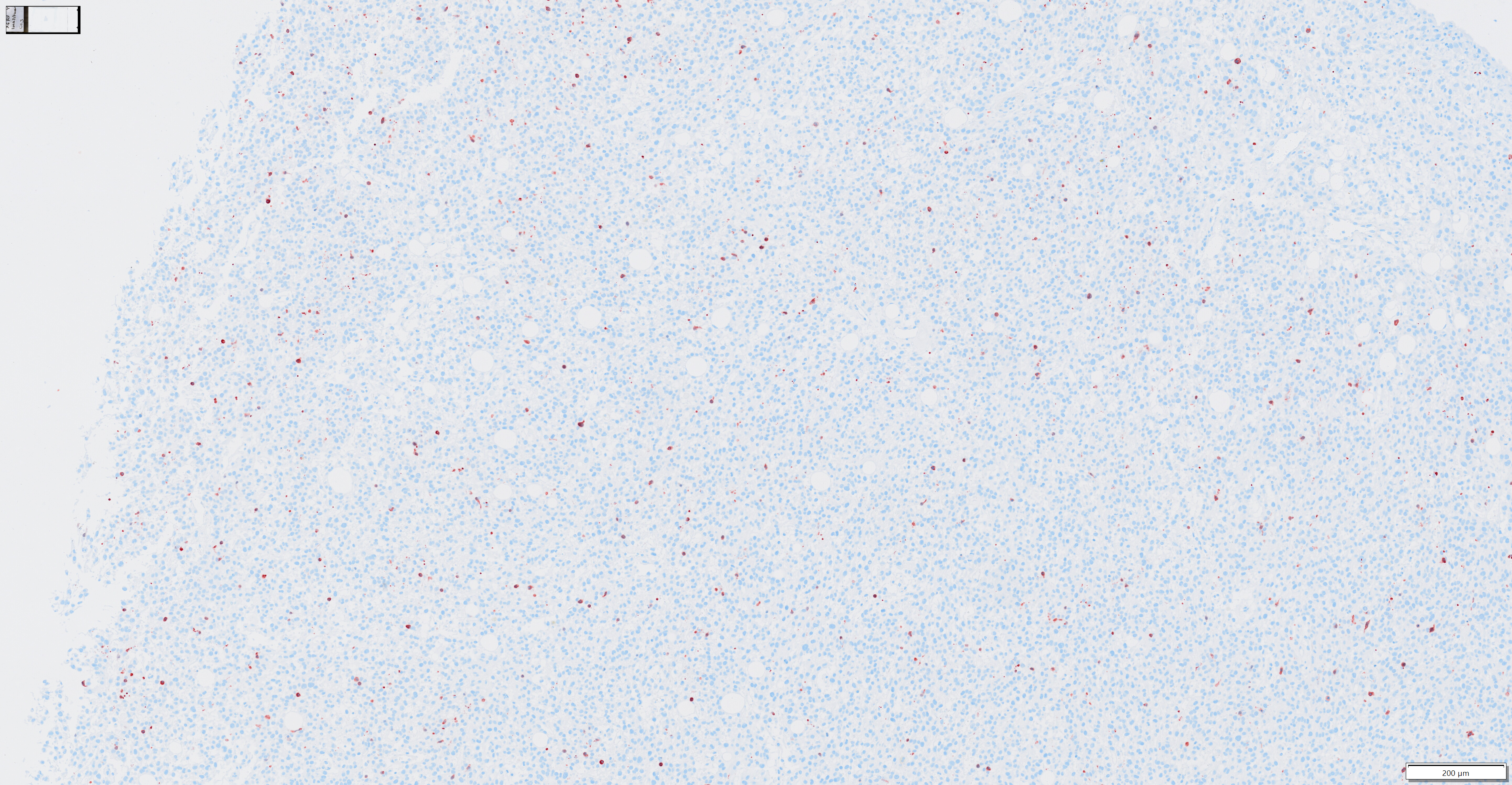

Supplement: Supplementary file 8 — Source data Fig. 3 [file 44318_2025_603_MOESM8_ESM.zip › EMBOJ2025120337_SourceData_Figure3/3I/Xenograft_Imatinib_CC3_200.tif]

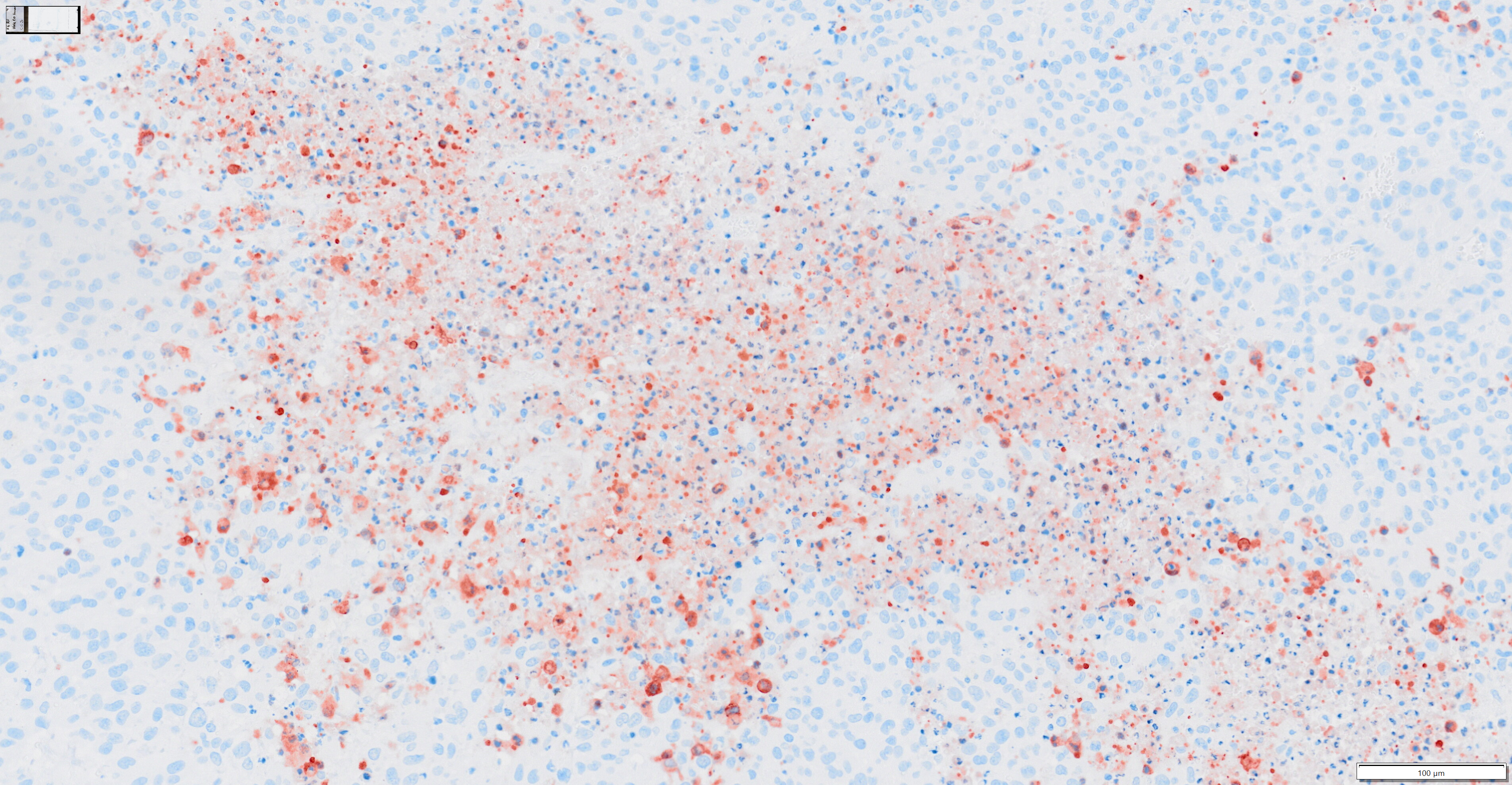

Supplement: Supplementary file 8 — Source data Fig. 3 [file 44318_2025_603_MOESM8_ESM.zip › EMBOJ2025120337_SourceData_Figure3/3I/Xenograft_Vehicle_CC3_100.tif]

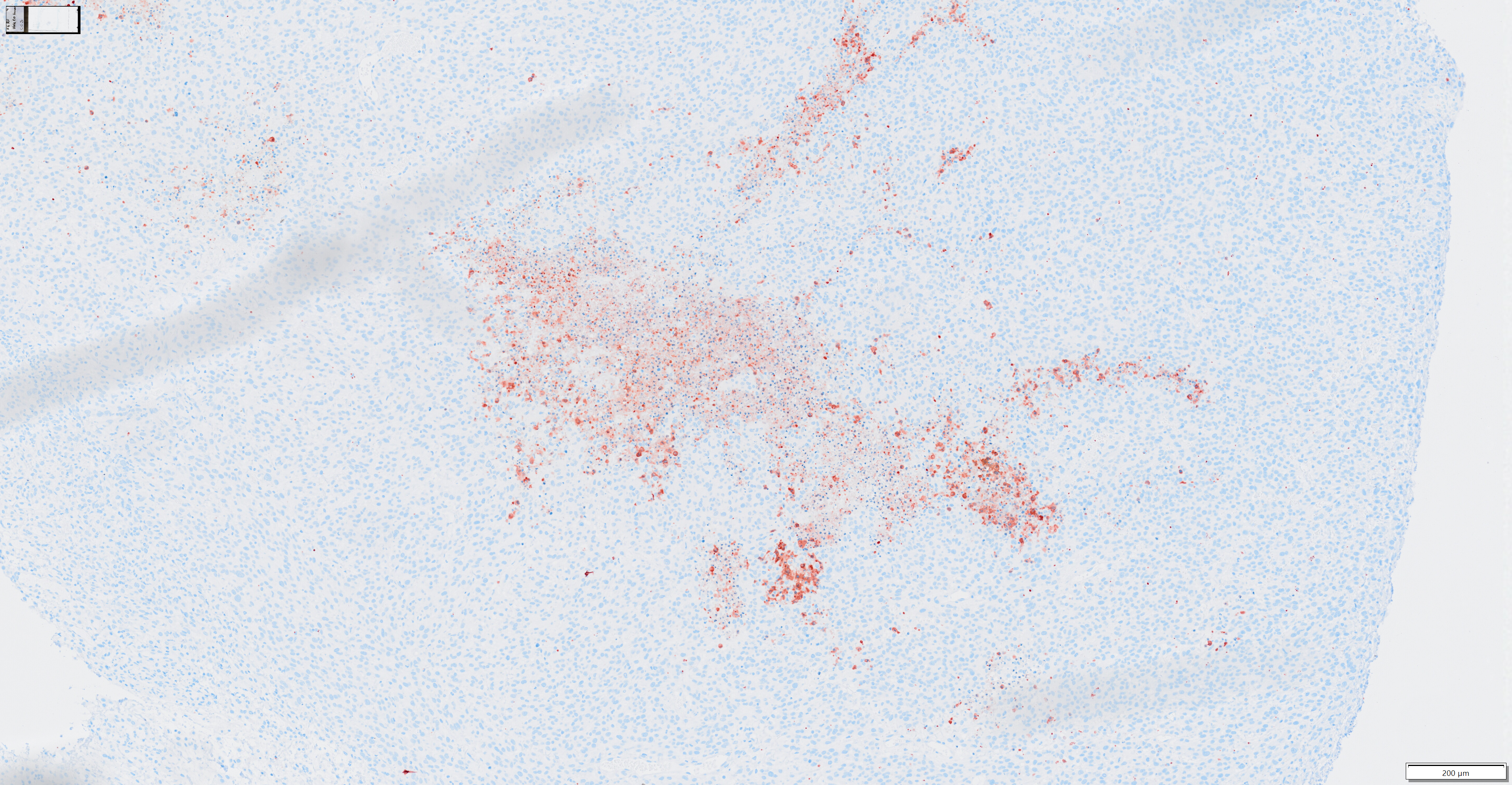

Supplement: Supplementary file 8 — Source data Fig. 3 [file 44318_2025_603_MOESM8_ESM.zip › EMBOJ2025120337_SourceData_Figure3/3I/Xenograft_Vehicle_CC3_200.tif]

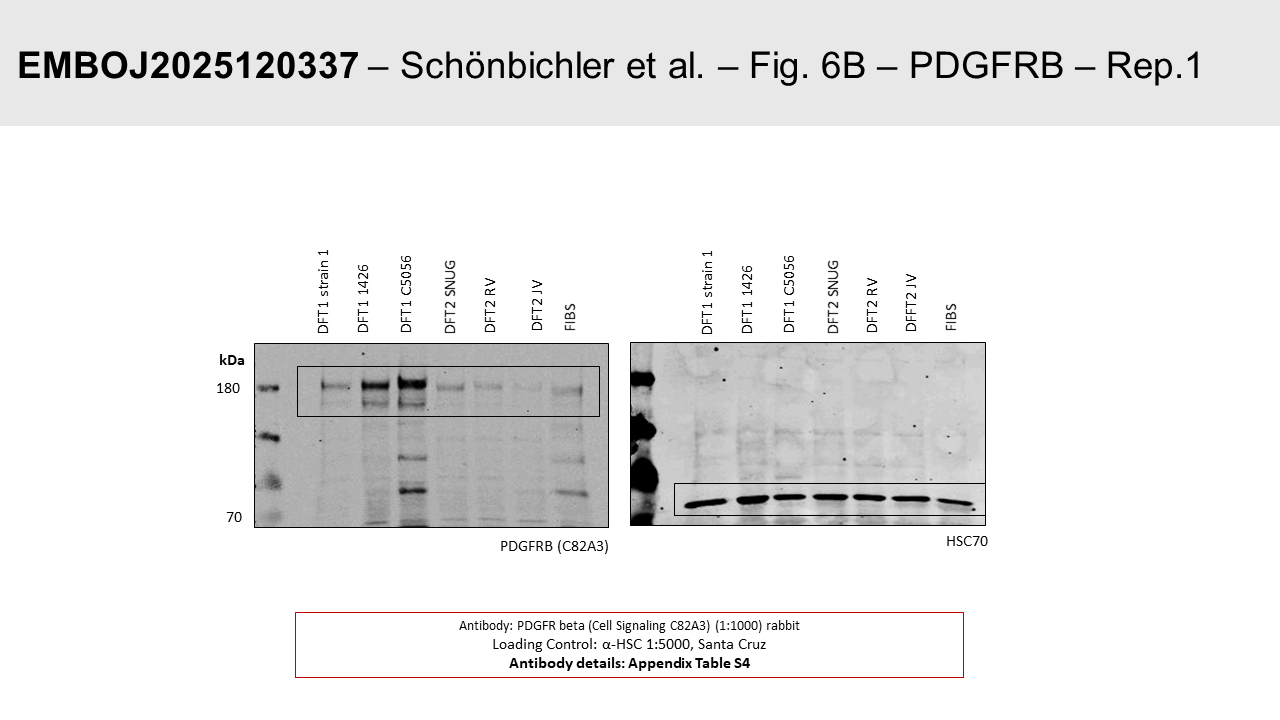

Supplement: Supplementary file 11 — Source data Fig. 6 [file 44318_2025_603_MOESM11_ESM.zip › EMBOJ2025120337_SourceData_Figure6/6B/PDGFRB_Western_Replicate1.tif]

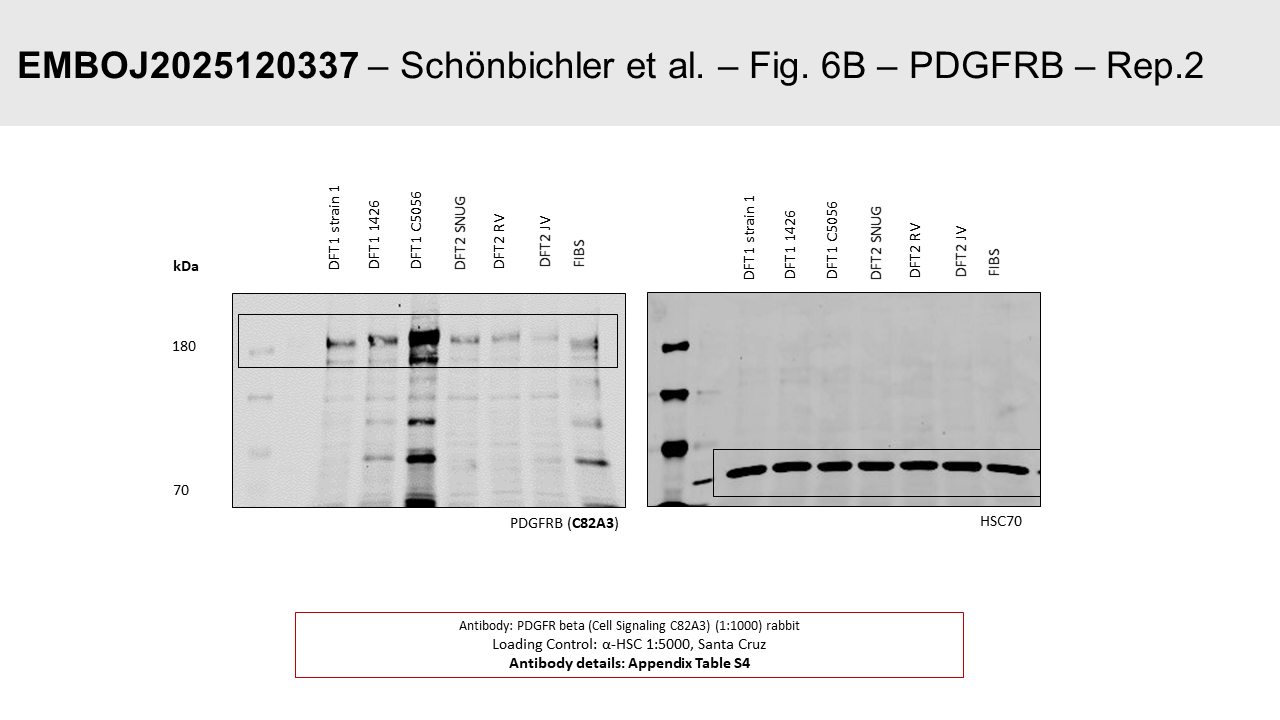

Supplement: Supplementary file 11 — Source data Fig. 6 [file 44318_2025_603_MOESM11_ESM.zip › EMBOJ2025120337_SourceData_Figure6/6B/PDGFRB_Western_Replicate2.tif]

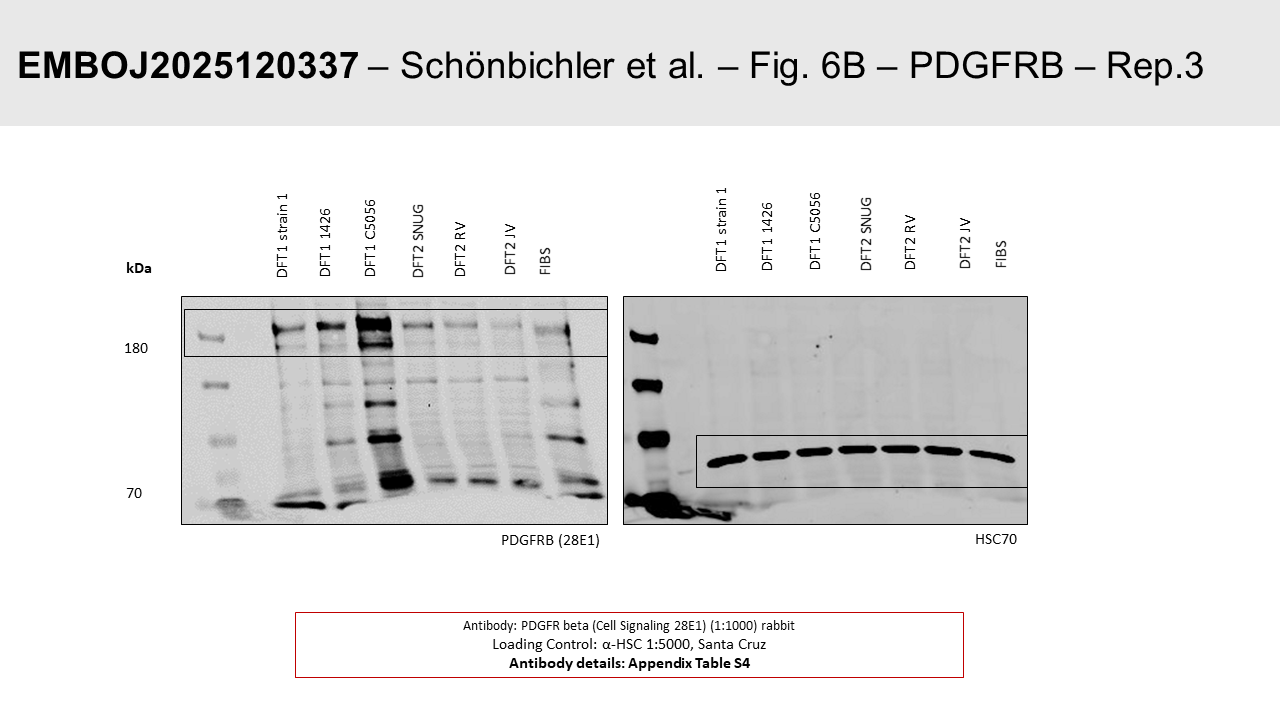

Supplement: Supplementary file 11 — Source data Fig. 6 [file 44318_2025_603_MOESM11_ESM.zip › EMBOJ2025120337_SourceData_Figure6/6B/PDGFRB_Western_Replicate3.tif]

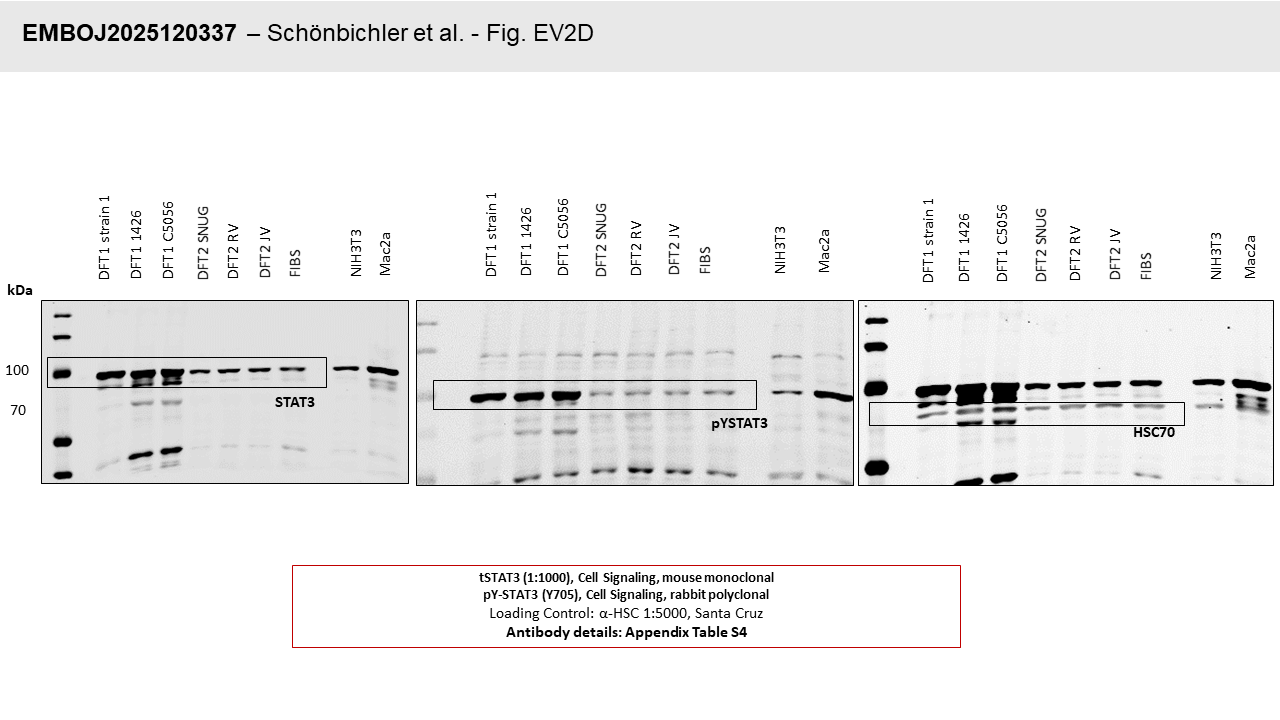

Supplement: Supplementary file 12 — Figure EV2 Source Data [file 44318_2025_603_MOESM12_ESM.zip › EMBOJ2025120337_SourceData_FigureEV2/EV2D/STAT3_pYSTAT3_western.tif]

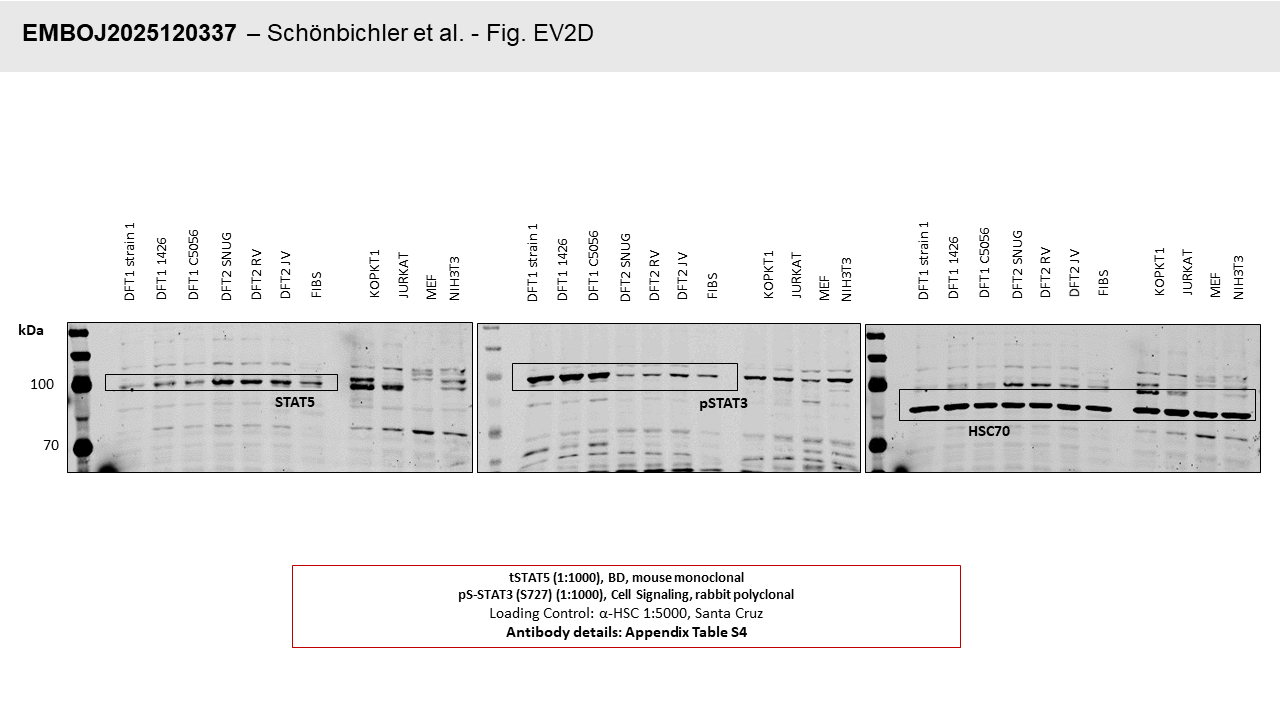

Supplement: Supplementary file 12 — Figure EV2 Source Data [file 44318_2025_603_MOESM12_ESM.zip › EMBOJ2025120337_SourceData_FigureEV2/EV2D/STAT5_pSSTAT3_western.tif]

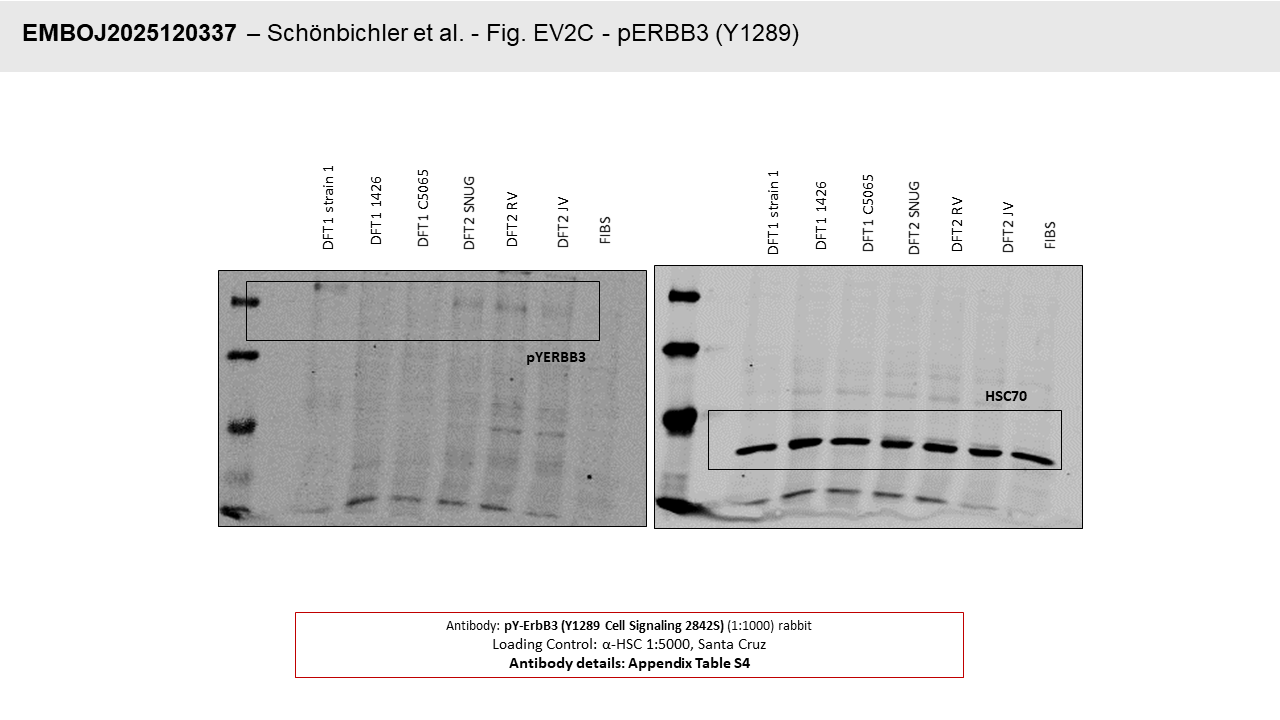

Supplement: Supplementary file 12 — Figure EV2 Source Data [file 44318_2025_603_MOESM12_ESM.zip › EMBOJ2025120337_SourceData_FigureEV2/EV2C/pERBB_western.tif]

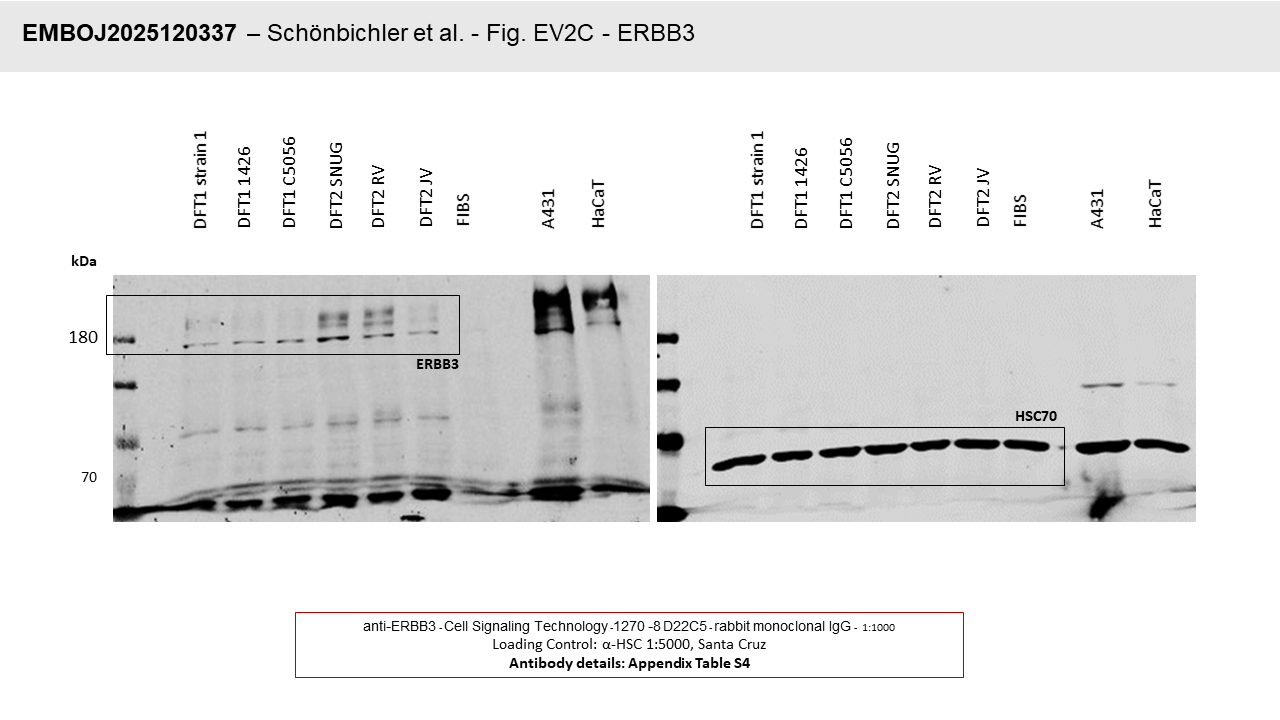

Supplement: Supplementary file 12 — Figure EV2 Source Data [file 44318_2025_603_MOESM12_ESM.zip › EMBOJ2025120337_SourceData_FigureEV2/EV2C/ERBB3_western.tif]

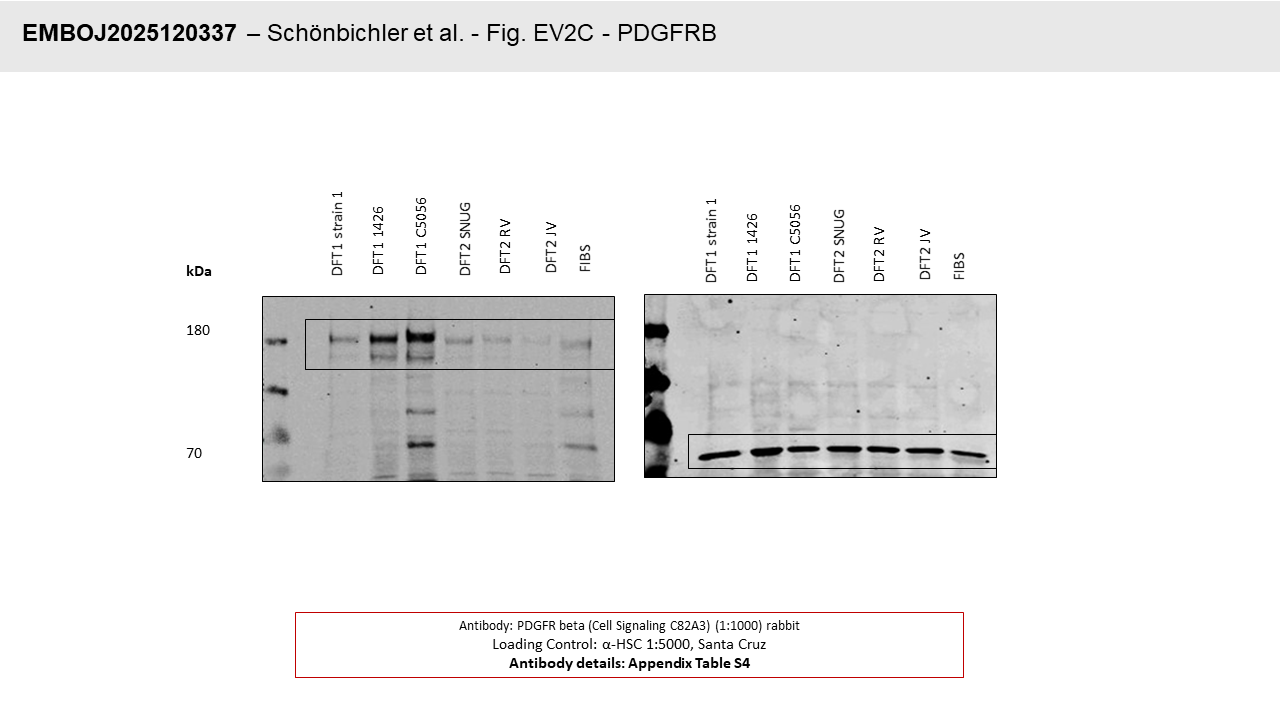

Supplement: Supplementary file 12 — Figure EV2 Source Data [file 44318_2025_603_MOESM12_ESM.zip › EMBOJ2025120337_SourceData_FigureEV2/EV2C/PDGFRB_western.tif]

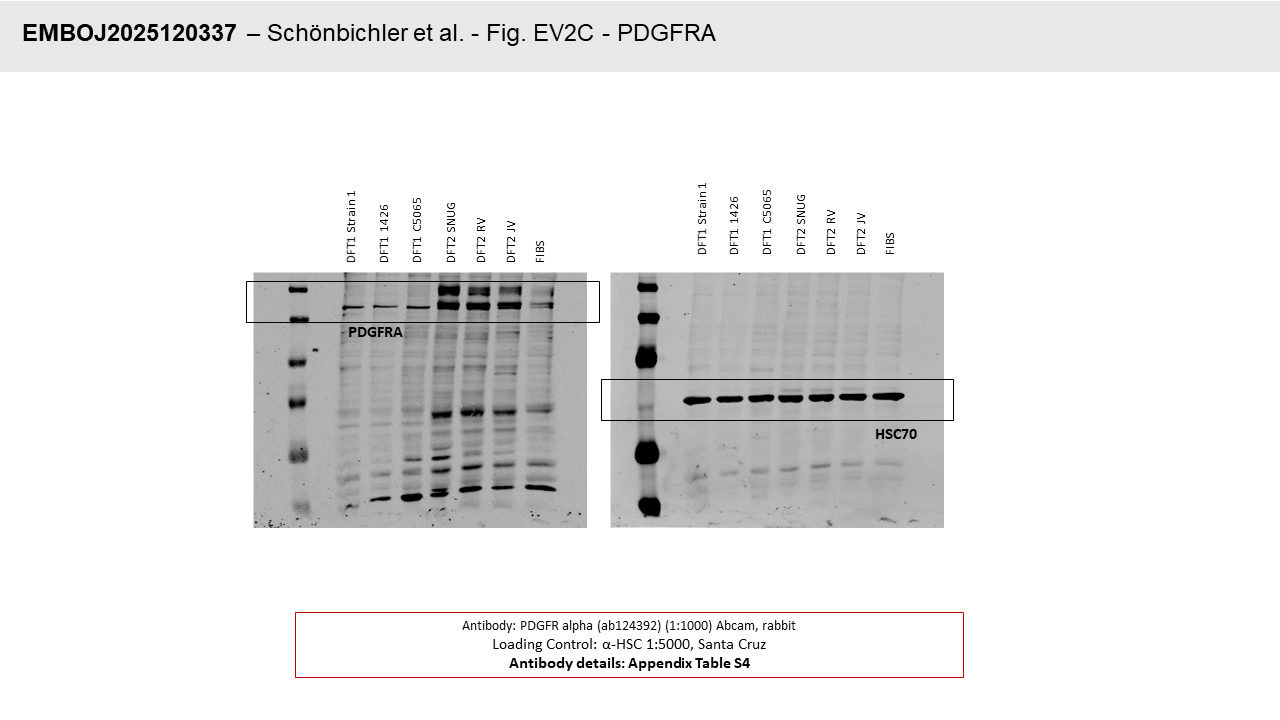

Supplement: Supplementary file 12 — Figure EV2 Source Data [file 44318_2025_603_MOESM12_ESM.zip › EMBOJ2025120337_SourceData_FigureEV2/EV2C/PDGFRA_western.tif]

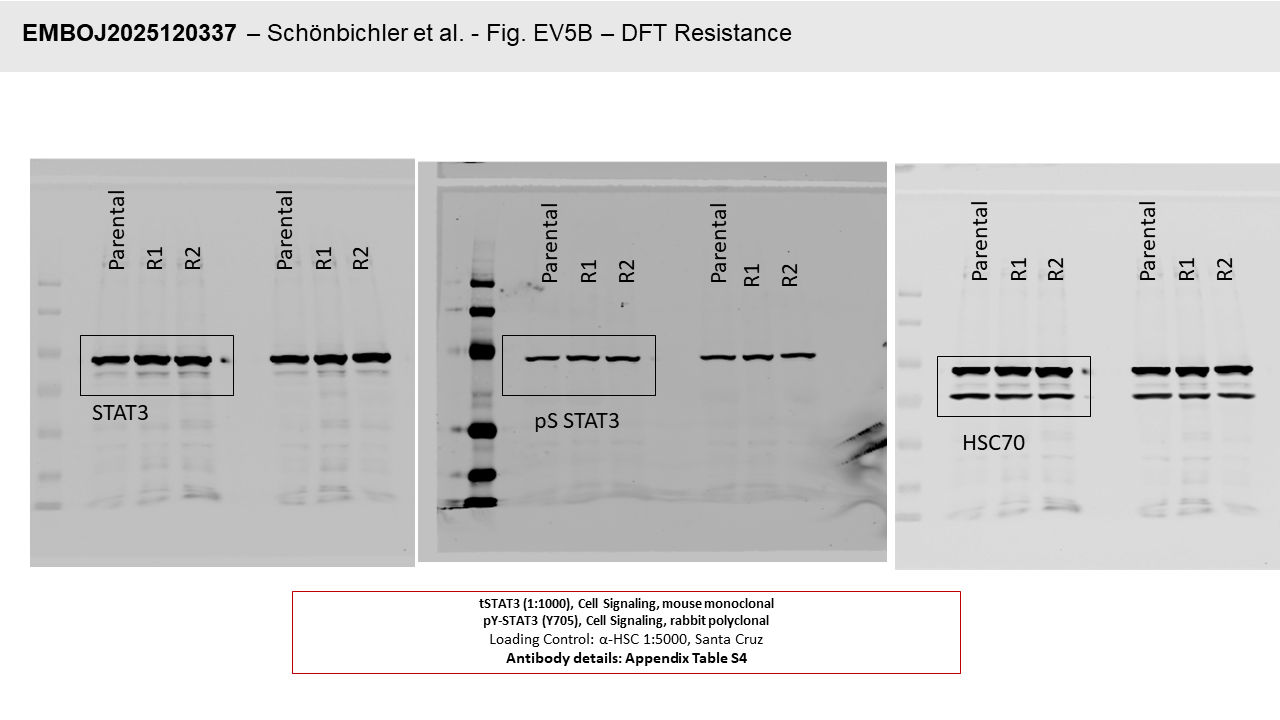

Supplement: Supplementary file 13 — Figure EV5 Source Data [file 44318_2025_603_MOESM13_ESM.zip › EMBOJ2025120337_SourceData_FigureEV5/EV5B/STAT3_pSTAT3_Resistance_DFT1_Western.tif]

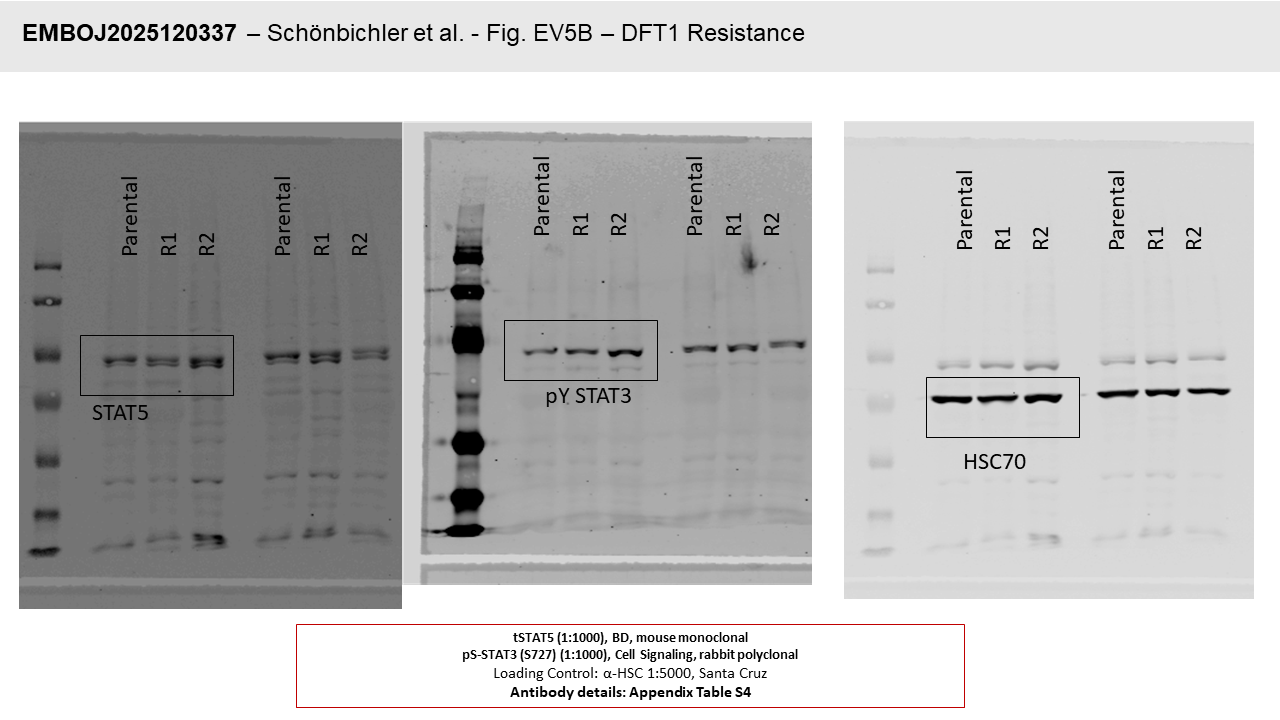

Supplement: Supplementary file 13 — Figure EV5 Source Data [file 44318_2025_603_MOESM13_ESM.zip › EMBOJ2025120337_SourceData_FigureEV5/EV5B/STAT5_pSTAT3_Resistance_DFT1_Western.tif]

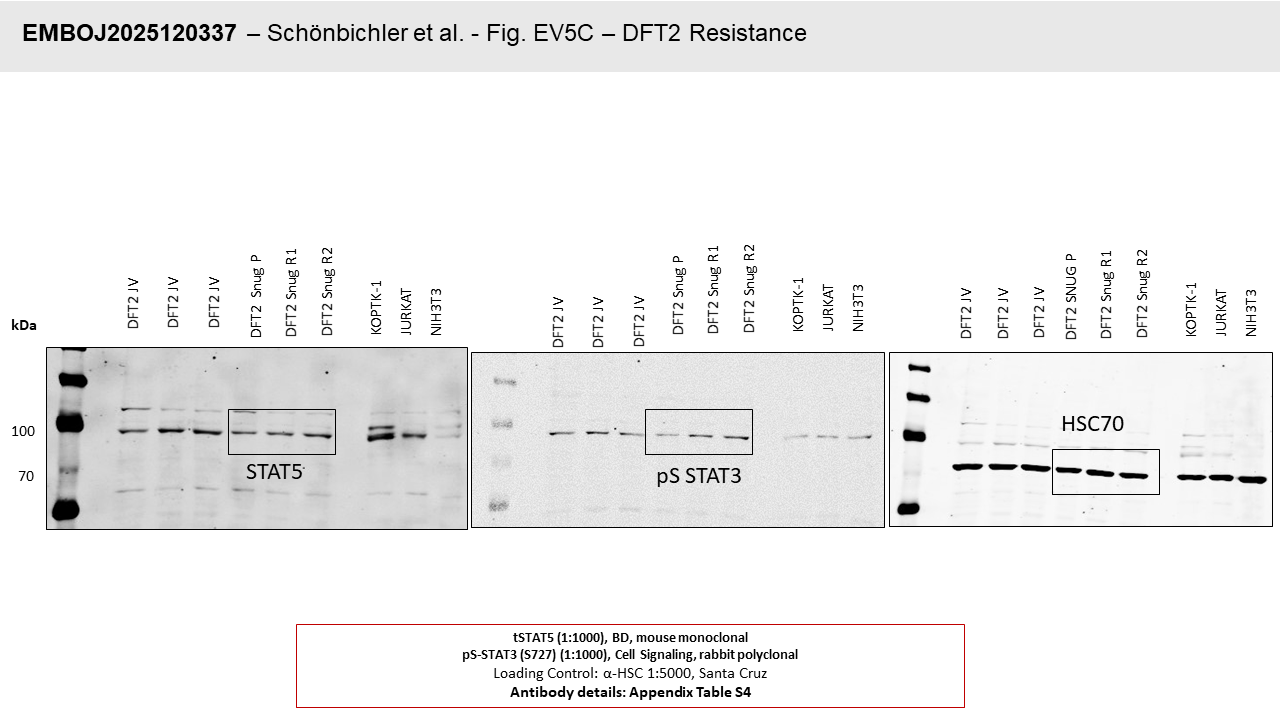

Supplement: Supplementary file 13 — Figure EV5 Source Data [file 44318_2025_603_MOESM13_ESM.zip › EMBOJ2025120337_SourceData_FigureEV5/EV5C/STAT5_pSTAT3_Resistance_DFT2_Western.tif]

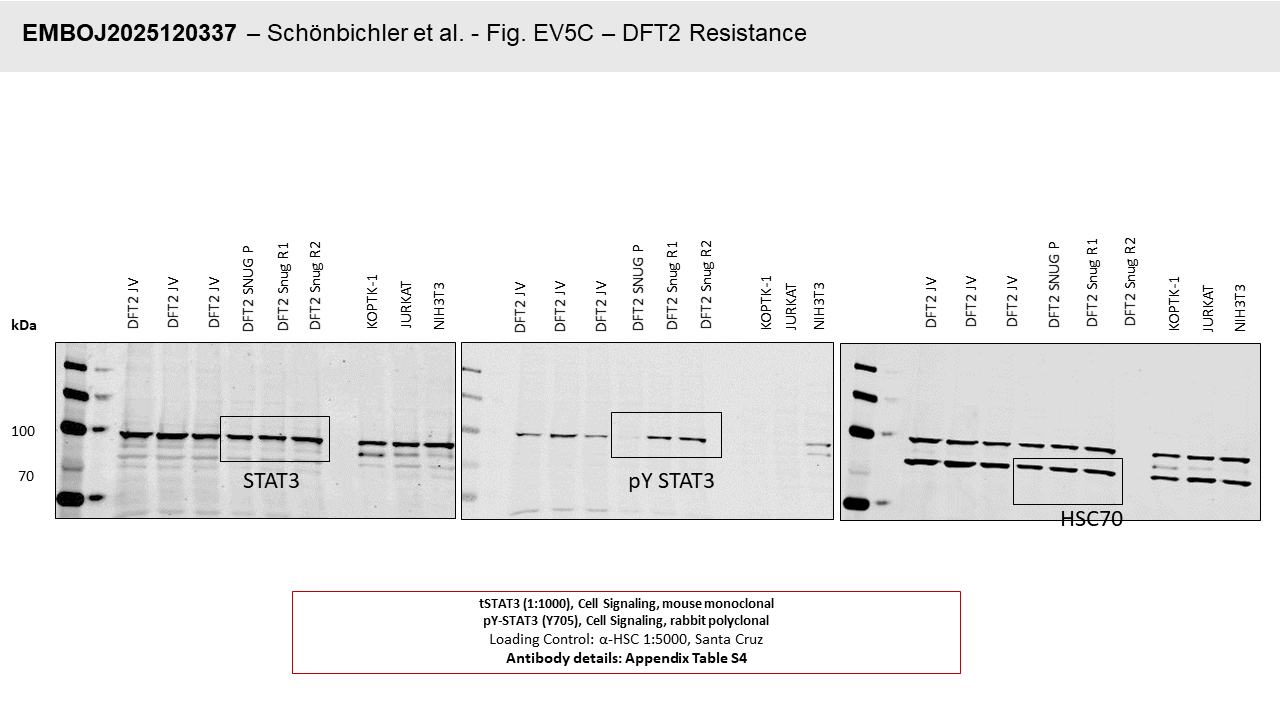

Supplement: Supplementary file 13 — Figure EV5 Source Data [file 44318_2025_603_MOESM13_ESM.zip › EMBOJ2025120337_SourceData_FigureEV5/EV5C/STAT3_pSTAT3_Resistance_DFT2_Western.tif]
