# Supplementary material for: Tyrosine kinase targeting uncovers oncogenic pathway plasticity in Tasmanian devil transmissible cancers
Source: EMBO J. 2025 Nov 3;45(5):1426–59. doi: 10.1038/s44318-025-00603-0 (PMC12953634; doi:10.1038/s44318-025-00603-0)
Supplement: Supplementary file 16 — Expanded View Figures [file 44318_2025_603_MOESM16_ESM.pdf]

Expanded View Figures

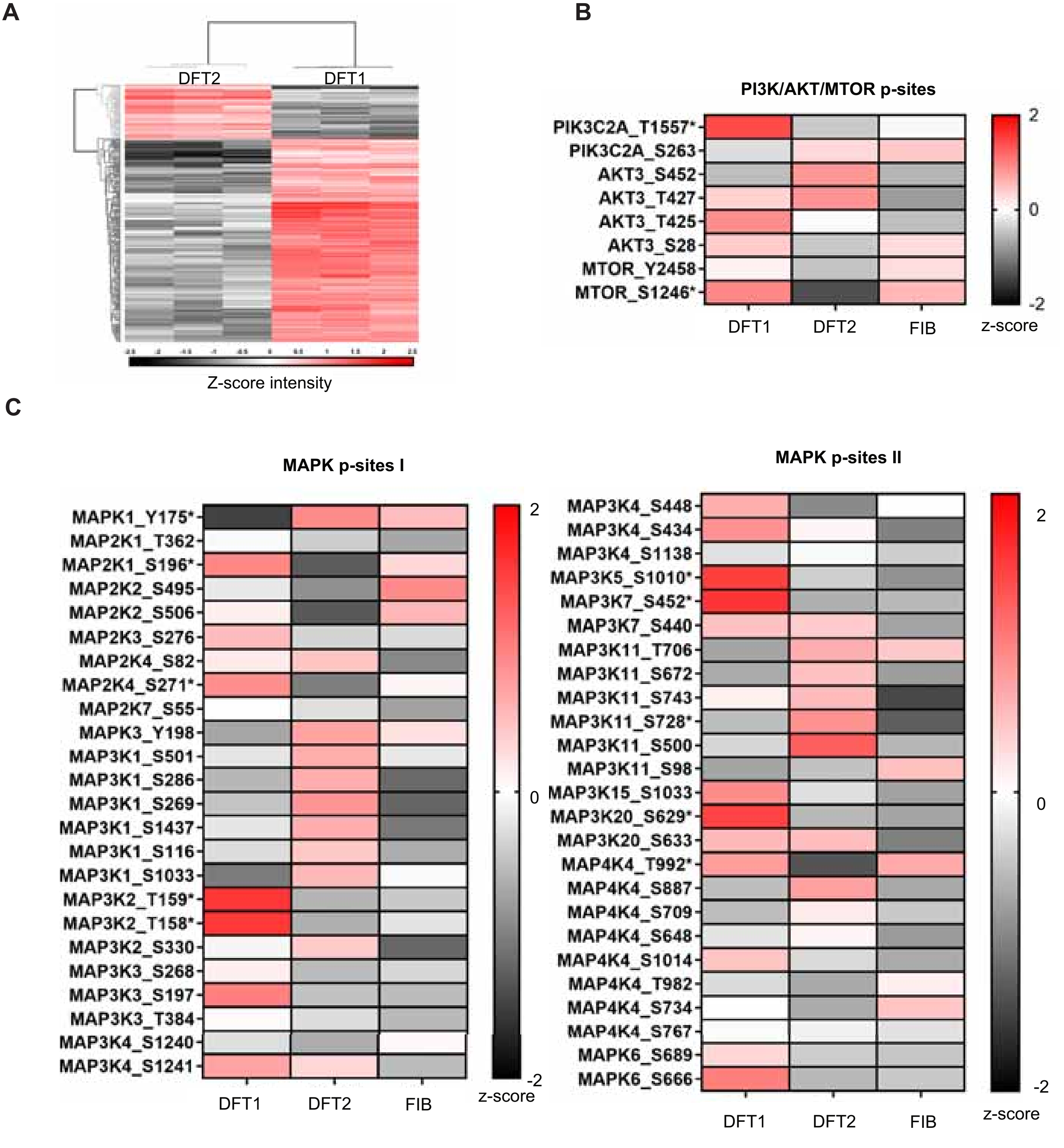

Figure EV1. Related to Fig. 1.

(**A**) Heatmap of hierarchical clustering of z-scored log2 intensity values for significantly differentially abundant phosphopeptides (Fig. 1C). (**B**, **C**) Heatmaps of relative abundance (z-scored log2 intensity values) for peptides mapped to PI3K/AKT/mTOR pathway and MAPK pathway proteins. Stars indicate significant phosphorylation differences between DFT1 and DFT2 (*t* test, FDR < 0.05, s0 = 0.1).

 © The Author(s)

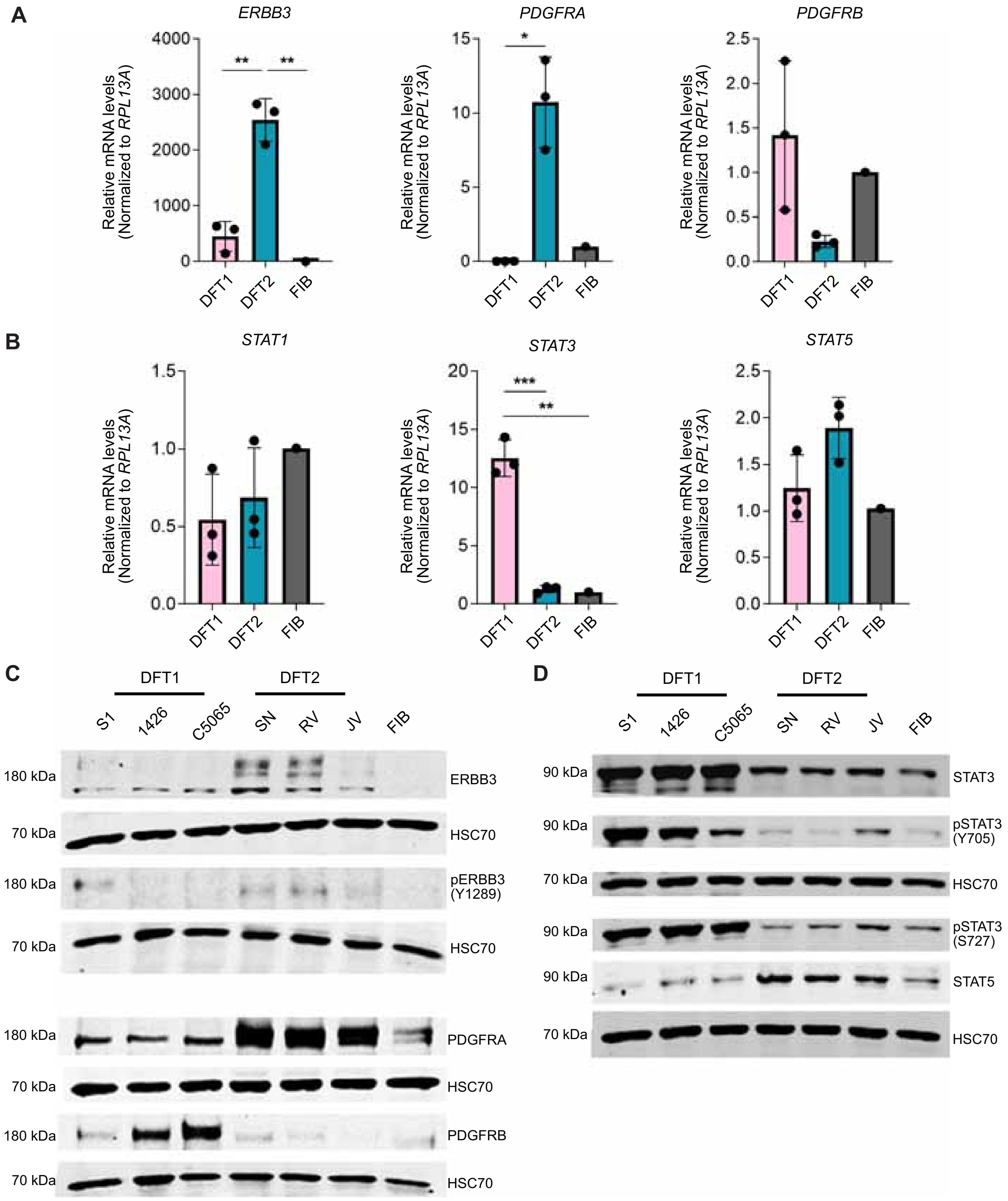
A
ERBB3
PDGFRA
PDGFRB
Relative mRNA levels (Normalized to RPL13A)
DFT1
DFT2
FIB
B
STAT1
STAT3
STAT5
C
DFT1
DFT2
S1
1426
C5065
SN
RV
JV
FIB
180 kDa
70 kDa
ERBB3
HSC70
pERBB3 (Y1289)
PDGFRA
PDGFRB
D
90 kDa
STAT3
pSTAT3 (Y705)
pSTAT3 (S727)
STAT5

Figure EV2. Related to Fig. 1.

(**A**, **B**) Gene expression of *ERBB3, PDGFRA, PDGFRB* and downstream signalling molecules *STAT1, STAT3, STAT5**) measured by qPCR and normalised to *RPL13A*. Data represent three DFT1 and three DFT2 cell lines (each dot corresponds to a cell line), shown as mean ± SD relative to a fibroblast control. Statistical analysis was performed using one-way ANOVA with Bonferroni's test. Statistical significance was set at $P < 0.05$ and is indicated as follows: $P < 0.0332$ (*), $P < 0.0021$ (**), $P < 0.0002$ (***), and $P < 0.0001$ (****). Exact P values were as follows: *ERBB3* – DFT1 vs. DFT2: $P = 0.0045$; DFT2 vs. FIB: $P = 0.008$. *PDGFRA* – DFT1 vs. DFT2: $P = 0.0110$. *STAT3* – DFT1 vs. DFT2: $P = 0.0008$; DFT1 vs. FIB: $P = 0.0028$. Data shown are from one representative experiment of two independent replicates. (**C**, **D**) Western blot analysis of ERBB3, pERBB3 (Y1289), PDGFRA, PDGFRB, STAT3, pSTAT3 (Y705), pSTAT3 (S727) and STAT5* in six DFT cell lines and one fibroblast control. HSC70 was the loading control. $n = 3$. **The Tasmanian devil genome contains two genes more homologous to human STAT5B than STAT5A, suggesting the absence of STAT5A protein*. Source data are available online for this figure.

 © The Author(s)

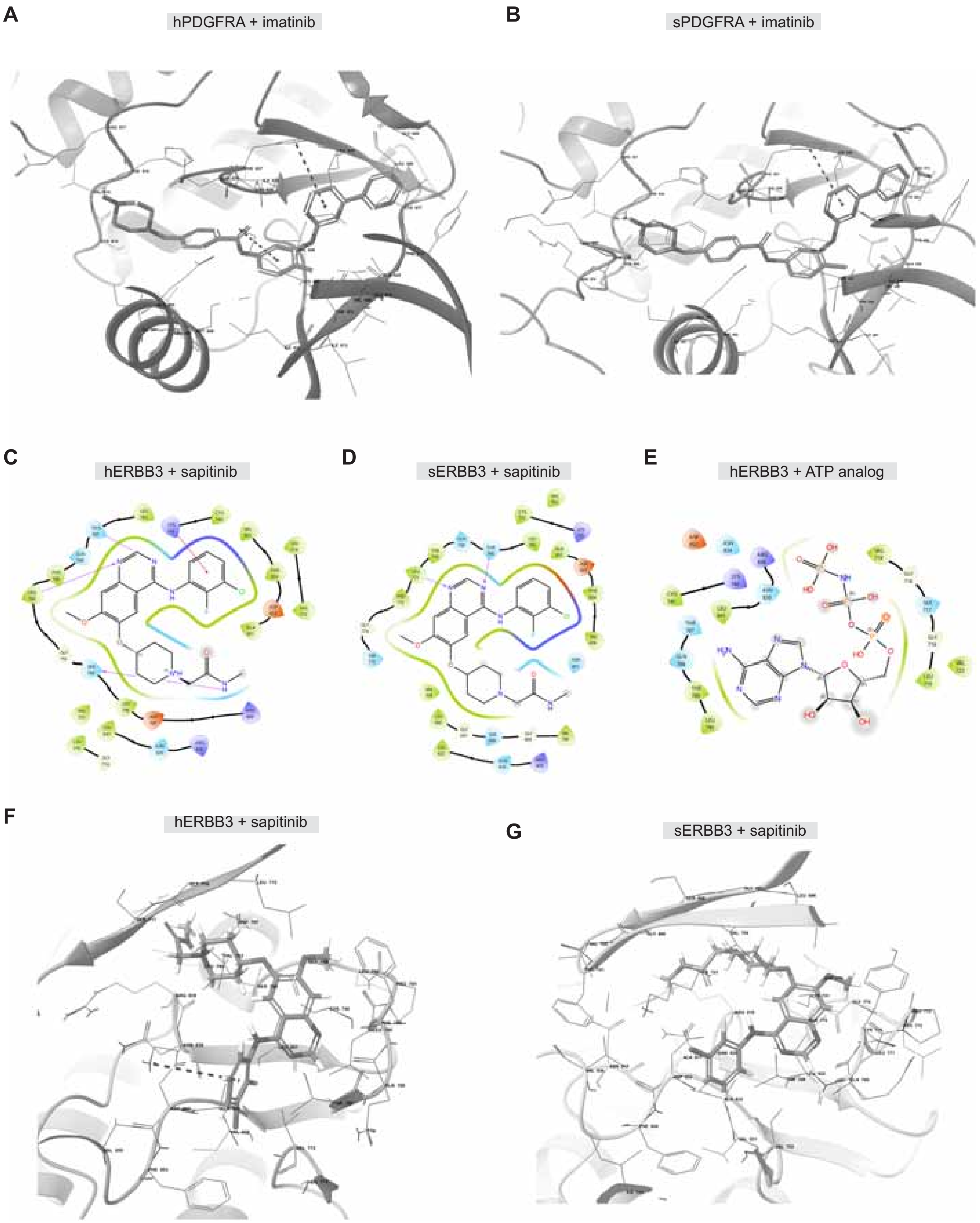
A
hPDGFRA + imatinib
B
sPDGFRA + imatinib
C
hERBB3 + sapitinib
D
sERBB3 + sapitinib
E
hERBB3 + ATP analog
F
hERBB3 + sapitinib
G
sERBB3 + sapitinib

Figure EV3. Associated with Fig. 2.

(**A**, **B**) Structural depiction of human PDGFRA (hPDGFRA, **A**) bound to imatinib (PDB ID: 6JOL) and Tasmanian devil PDGFRA (sPDGFRA, **B**) modeled into the 6JOL structure, showing binding poses and interacting residues at the catalytic site of the kinase domain. (**C**–**E**) Residues of human ERBB3 (hERBB3, **C**) and Tasmanian devil ERBB3 (sERBB3, **D**) predicted to interact with sapitinib at the catalytic site of the kinase domain. For comparison, residues involved in binding an ATP analog ligand in the hERBB3 crystal structure (PDB ID: 4RIX) are shown (**E**). Note that residues in the 4RIX structure are truncated at the N-terminus. (**F**, **G**) Induced-fit docking of sapitinib with hERBB3 (**F**) and sERBB3 (**G**), illustrating predicted binding poses and interacting residues at the catalytic site.

© The Author(s)

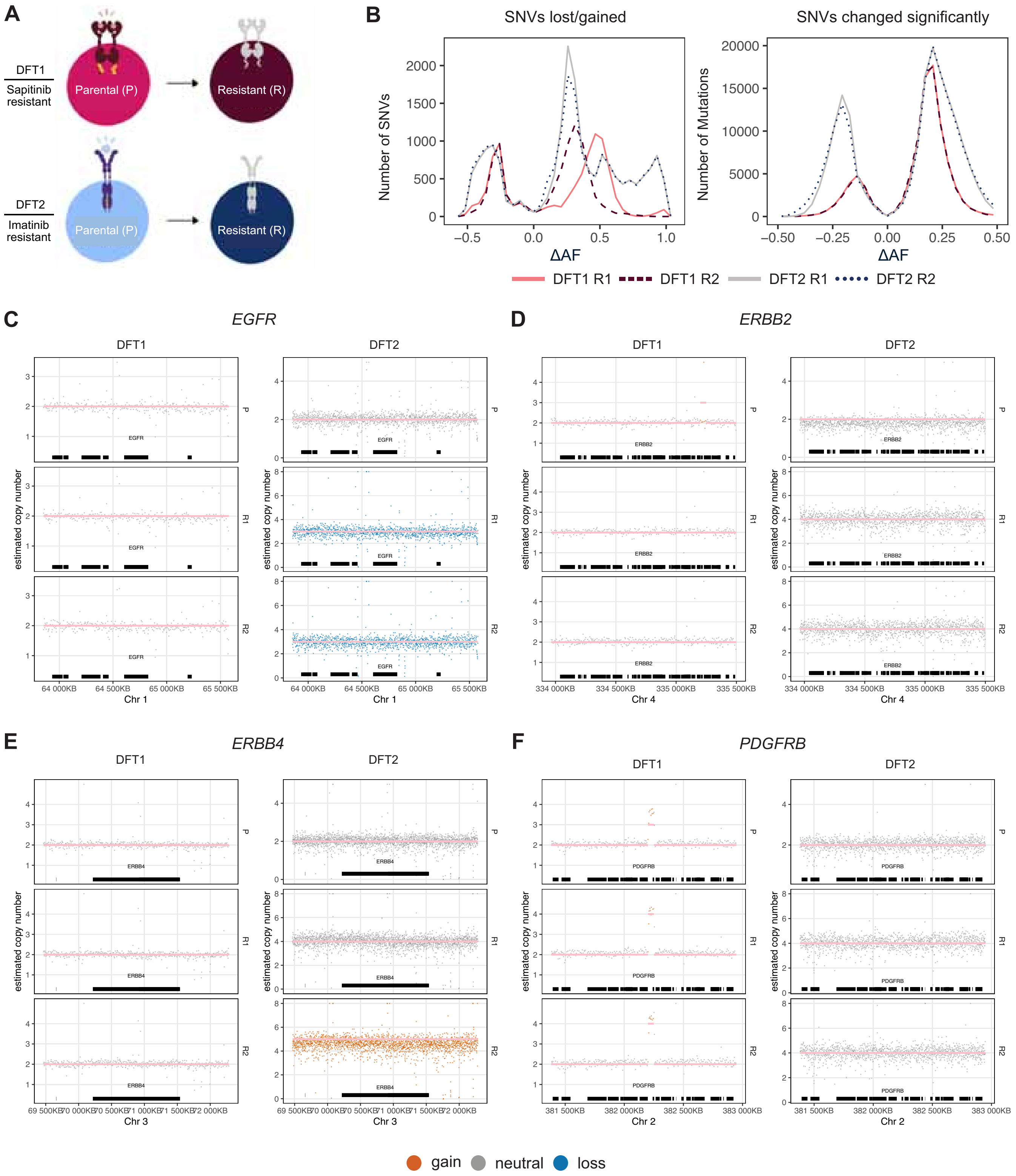
A
DFT1
Sapitinib resistant
Parental (P)
Resistant (R)
DFT2
Imatinib resistant
B
SNVs lost/gained
SNVs changed significantly
Number of SNVs
Number of Mutations
ΔAF
DFT1 R1
DFT1 R2
DFT2 R1
DFT2 R2
C
EGFR
D
ERBB2
E
ERBB4
F
PDGFRB
DFT1
DFT2
estimated copy number
P
R1
R2
Chr 1
Chr 2
Chr 3
Chr 4
gain
neutral
loss

Figure EV4. Associated with Fig. 4.

(**A**) Schematic showing the generation of sapitinib-resistant DFT1 and imatinib-resistant DFT2 cell lines. (**B**) Distribution of SNVs gained or lost in resistant lines. Left panel: ΔAF represents the change in minor allele frequency relative to the parental line. SNVs with ΔAF < 0 were considered lost; ΔAF > 0 indicates novel SNVs. Y-axis shows the number of mutations at each ΔAF bin. Filtering was performed using an FDR-corrected binomial test ($P < 0.1$). Right panel: SNVs showing statistically significant frequency changes between parental and resistant lines, identified using Fisher's exact test ($P < 0.05$). (**C–F**) Copy number (CN) profiles for *EGFR, ERBB2, ERBB4* and *PDGFRB. Normalised* CN rations were calculated by Control-FREEC using 5 kbp or 1 kbp genomic windows for DFT1 and DFT2 respectively and multiplied by the assumed ploidy. Genes are indicated as black bars. Copy number gains are shown in orange and losses in blue. Data are displayed in 5 kbp or 1 kbp windows. The pink line represents estimated copy number inferred by Control-FREEC.

 © The Author(s)

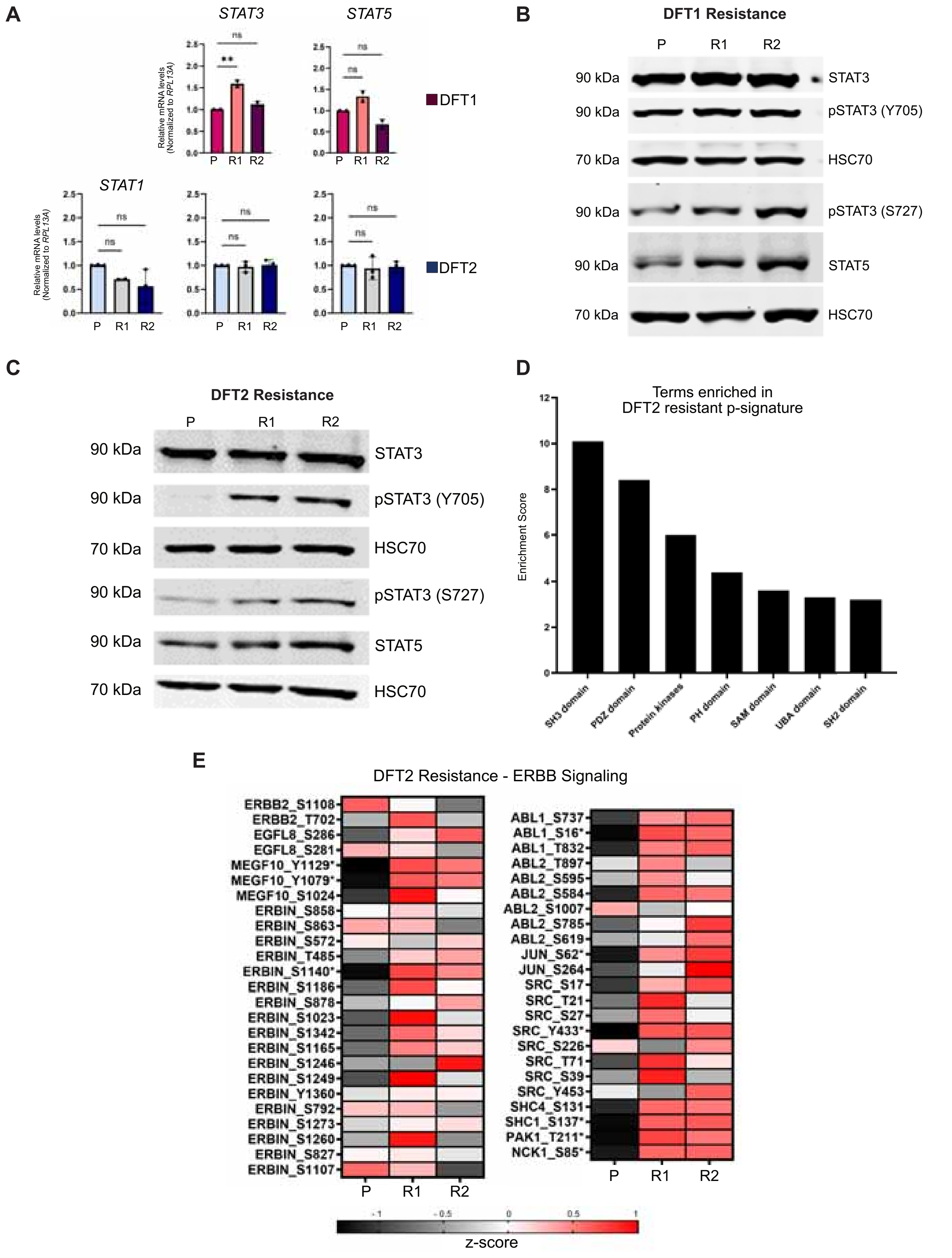
A
STAT3
STAT5
DFT1
DFT2
STAT1
Relative mRNA levels (Normalized to RPL13A)
P R1 R2
ns
**
B
DFT1 Resistance
P R1 R2
90 kDa STAT3
90 kDa pSTAT3 (Y705)
70 kDa HSC70
90 kDa pSTAT3 (S727)
90 kDa STAT5
70 kDa HSC70
C
DFT2 Resistance
P R1 R2
90 kDa STAT3
90 kDa pSTAT3 (Y705)
70 kDa HSC70
90 kDa pSTAT3 (S727)
90 kDa STAT5
70 kDa HSC70
D
Terms enriched in DFT2 resistant p-signature
Enrichment Score
SH3 domain
PDZ domain
Protein kinases
PH domain
SAM domain
UBA domain
SH2 domain
E
DFT2 Resistance - ERBB Signaling
ERBB2_S1108
ERBB2_T702
EGFL8_S286
EGFL8_S281
MEGF10_Y1129*
MEGF10_Y1079*
MEGF10_S1024
ERBIN_S858
ERBIN_S863
ERBIN_S572
ERBIN_T485
ERBIN_S1140*
ERBIN_S1186
ERBIN_S878
ERBIN_S1023
ERBIN_S1342
ERBIN_S1165
ERBIN_S1246
ERBIN_S1249
ERBIN_Y1360
ERBIN_S792
ERBIN_S1273
ERBIN_S1260
ERBIN_S827
ERBIN_S1107
ABL1_S737
ABL1_S16*
ABL1_T832
ABL2_T897
ABL2_S595
ABL2_S584
ABL2_S1007
ABL2_S785
ABL2_S619
JUN_S62*
JUN_S264
SRC_S17
SRC_T21
SRC_S27
SRC_Y433*
SRC_S226
SRC_T71
SRC_S39
SRC_Y453
SHC4_S131
SHC1_S137*
PAK1_T211*
NCK1_S85*
P R1 R2
z-score

Figure EV5. Associated with Fig. 5.

(**A**) Gene expression of *STAT1, STAT3*, and *STAT5* in parental (P) and resistant (R1, R2) DFT1 (top) and DFT2 (bottom) cell lines, quantified by real-time PCR and normalised to *RPL13A*. Data are shown relative to the respective parental control and presented as mean ± SD ($n = 3$ replicates). Statistical analysis was performed using one-way ANOVA with Bonferroni's multiple comparisons test, comparing each group to the parental (P) group. Statistical significance was set at $P < 0.05$ and is indicated as follows: $P < 0.0332$ (*), $P < 0.0021$ (**), $P < 0.0002$ (***), and $P < 0.0001$ (****). STAT3 (DFT1) P vs. R1; $P = 0.0058$. (**B**, **C**) Western blot analysis of STAT3, pSTAT3 (Y705), pSTAT3 (S727), and STAT5 in parental and resistant DFT1 (**B**) and DFT2 (**C**) cell lines. HSC70 served as a loading control. $n = 2$. (**D**) Bar plot showing annotation terms enriched in the DFT2 resistant p-peptide signature. Terms were determined by functional annotation clustering using DAVID Bioinformatics, with selection based on enrichment score <3 and *P* value < 0.05 (Bonferroni correction). (**E**) Heatmaps of the relative abundance (z-scored log2 intensity values) of ERBB pathway p-peptides in DFT2 parental and imatinib-resistant cell lines. *Significant differences in phosphorylation between parental and resistant cells lines (*P* value < 0.05). Source data are available online for this figure.

 © The Author(s)
